# Systemic delivery of stealth adenoviral vector to reinvigorate antitumor immunity by PD-L1 genome editing and TIGIT/CD155 blocking

**DOI:** 10.1016/j.fmre.2025.04.015

**Published:** 2025-05-14

**Authors:** Ming Wu, Cao Zhang, Aoxue Zhong, Hao Li, Yang Zhou, Xiaoyuan Zheng, Youshi Zheng, Yuting Zhang, Yanbing Cao, Zhenli Li, Cuilin Zhang, Xiaomo Wu, Dong Cen, Xiaolong Liu

**Affiliations:** a The United Innovation of Mengchao Hepatobiliary Technology Key Laboratory of Fujian Province, Mengchao Hepatobiliary Hospital of Fujian Medical University, Fuzhou 350025, China; b The Liver Center of Fujian Province, Fujian Medical University, Fuzhou 350025, China; c Mengchao Med-X Center, Fuzhou University, Fuzhou 350116, China; d Dermatology Institute of Fuzhou, Dermatology Hospital of Fuzhou, Fuzhou 350025, China; e Department of General Surgery, Sir Run Shaw Hospital, Zhejiang University School of Medicine, Hangzhou 310016, China

**Keywords:** Adenoviral vector, Genetically engineered cell membrane, PD-L1 genome editing, TIGIT/CD155 blockade, Cancer immunotherapy

## Abstract

Treatment with combined immune checkpoint blockade (CICB) has been considered as an effective approach to increase the objective response rate for cancer immunotherapy, but at the cost of significantly increased immune-related adverse events (irAEs) and also sufferings from drug resistance due to the transitory working manner of antibody-based ICB inhibitors. We introduce an antibody-independent CICB strategy using an adenoviral vector (sgCas9-Adv@TCM) that delivers a PD-L1 targeted CRISPR/Cas9 system, with a cell membrane cloak presenting TIGIT proteins. The vector could effectively accumulate in tumors, allowing permanent PD-L1 genome editing and TIGIT/CD155 blocking through systemic administration while evading detection and degradation by the immune system. The sgCas9-Adv@TCM vector achieved complete tumor regression in 80% of mice in a subcutaneous hepatocellular carcinoma (HCC) model by globally reshaping the tumor immune microenvironment to elicit robust anti-tumor immunity. Furthermore, sgCas9-Adv@TCM was proven to be still competent and even superior to the standard FDA-approved CICB regimen of the combined anti-PD-L1 and anti-TIGIT in suppressing tumor progression, prolonging survival, and inducing immunological memory of mice in the orthotopic HCC model, but with a much lower risk of irAEs. In addition, the clinical translation potential of sgCas9-Adv@TCM was further validated in the patient-derived organoid (PDO) and patient-derived xenograft (PDX) models from patients diagnosed with HCC. Thus, this study presents a safe and highly efficient “stealth” adenoviral vector with improved CICB features for cancer treatment.

## Graphical abstarct

**Figure:**
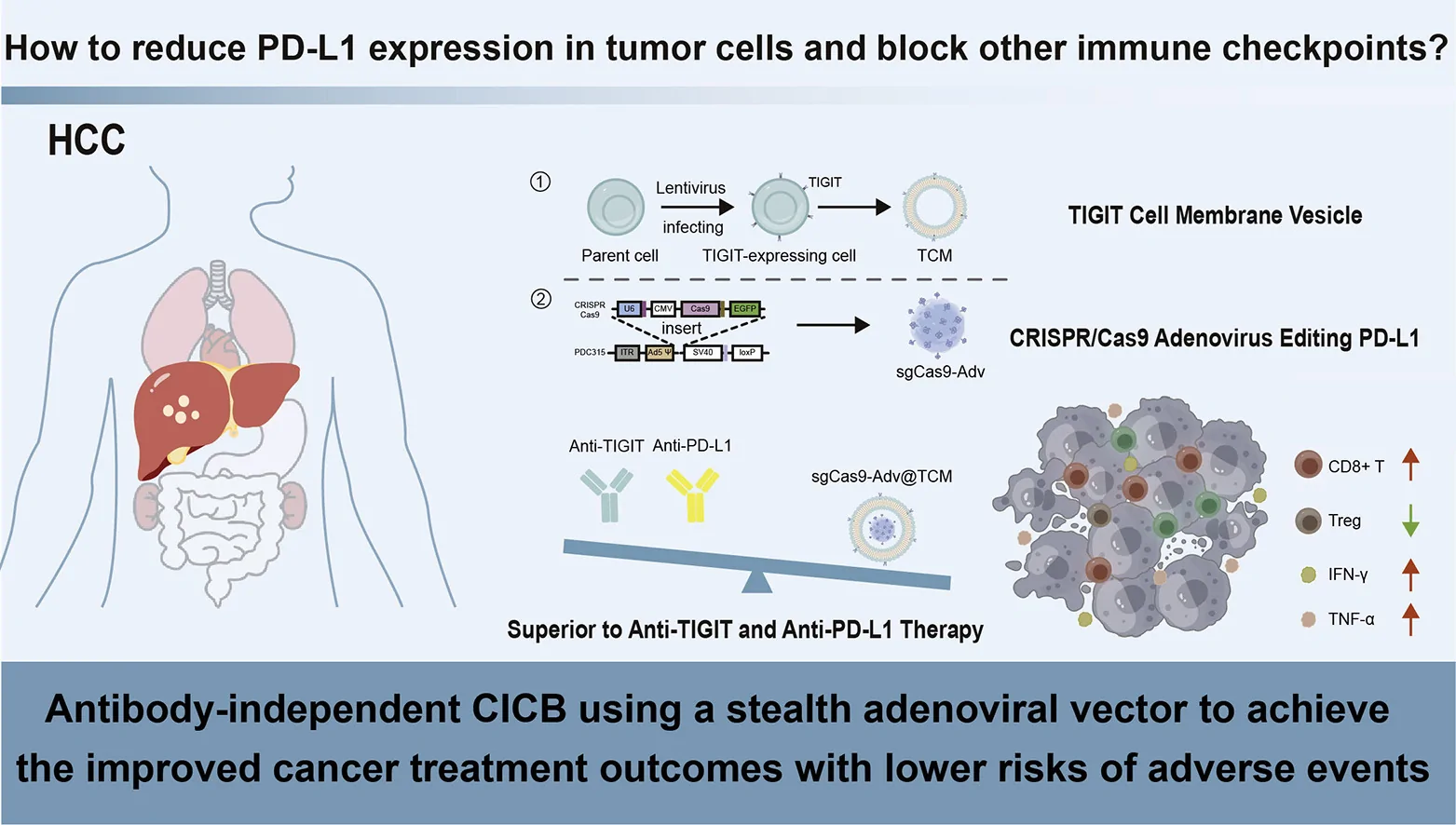

## 1 Introduction

Immune checkpoint blockade (ICB) therapy has made an indelible mark on the field of cancer treatment by unleashing the patient’s own immune system to kill cancer cells [1]. In particular, the use of monoclonal antibodies targeting inhibitory molecules of CTLA-4 and PD-1/PD-L1 is revolutionizing the oncology landscape due to their ability to induce unprecedented durable responses in cancer patients compared to other treatment approaches [2,3]. Nevertheless, due to the multiple mechanisms harnessed by cancer cells to evade immune surveillance, only a limited proportion of patients responded satisfactorily to the current single-agent ICB treatment [4]. Therefore, the preclinical and early clinical activities have been explored to combine various ICB inhibitors to improve therapeutic efficacy, but often at the expense of exacerbating side effects [[5], [6], [7]]. Meanwhile, drug resistance caused by the nature of the antibody itself can also minimize the therapeutic benefit. For instance, recent findings revealed that PD-L1 on the surface of tumor cells could be endogenously replenished and translated to the cell membrane even when blocked by PD-L1 antibodies [8,9]. Thus, there is a clear requirement to develop a highly effective combinational immune checkpoint blockade (CICB) strategy without exacerbating drug resistance or immune-related toxicity.

CRISPR-based genome-editing tools are becoming a powerful and reliable strategy for the targeted disruption of immune checkpoints in a permanent and precise manner, which shows great promise in overcoming the drug resistance due to the recycling of extracellular expression of PD-L1 harnessed by cancer cells [10]. In this regard, we recently developed an adenoviral vector-delivered PD-L1-targeted CRISPR/Cas9 system to enhance ICB efficacy in multiple tumor models due to its high gene transduction efficiency [11]. However, due to its severe immunogenicity, most studies, including ours and even other clinical trials, have to adopt local administration for adenovirus (Adv) based gene therapy [[11], [12], [13]]. However, most late-stage refractory tumors have inaccessible, disseminated, or metastatic characteristics, suggesting that systemic rather than localized delivery of Adv vectors should be more suitable to control cancer progression [14]. To date, systemic Adv delivery systems have primarily relied on the genetic reconstruction of the protein capsid, which is laborious and may also affect virus activity [14]. While another economic and practical approach is to build up a hybrid vector system by surface decoration of Adv with synthetic materials, such as polymers, liposomes, or nanoparticles [15], many of these materials cannot meet the clinical expectations as foreign substances due to the lack of biocompatibility or highly complex biological interactions in vivo.

In recent years, nature-inspired biomimetic nanocarriers have attracted significant attention for cancer treatment. Among them, coating nanoparticles with cell membranes/extracellular vesicles was employed to improve their biocompatibility and other in vivo performances [[16], [17], [18]]. The feasibility of this strategy has also been extended to viral decoration for immune evasion. For instance, a cell membrane cloaked oncolytic adenovirus displayed enhanced therapeutic efficacy compared to naked virus, with the merits of shielding the virus from neutralizing antibodies and a much lower risk of irAEs [19,20]. In addition to endowing Adv with natural surface properties and functions inherited from the sourced cells, cell membranes also can be genetically modified to provide application-specific functions, such as specific membrane protein anchoring for targeted delivery [21], immune checkpoint associated ligand/receptor/antibody displaying for immune regulations [22,23], and specific antigen presentation as vaccines [24].

T-cell immunoglobulin and ITIM domain (TIGIT), an inhibitory receptor expressed on lymphocytes, interacts with CD155 on tumor cells and tumor-associated myeloid cells to impair the function of T cells and natural killer (NK) cells [5,25]. As a novel ICB target, TIGIT/CD155 mediates immunosuppression in a manner similar to the PD-1/PD-L1 pathway. Currently, anti-TIGIT monoclonal antibodies (either alone or in combination with other ICB inhibitors) are being evaluated in ongoing Phase I-III trials to improve antitumor immune response. However, recent results from the Phase II trials showed that the co-formulation of vibostolimab (anti-TIGIT) and perbrolizumab (anti-PD-1) did not reach their primary endpoint [26]. Although it remains largely unclear what mechanisms are causing these unexpectedly negative studies, we hypothesize that an antibody-independent formulation that also targets both the PD-1/PD-L1 and TIGIT/CD155 pathways may be a promising substitute to enhance their combination efficacy. This approach could overcome the drug resistance and immune-related adverse events associated with antibodies, as discussed above.

As a proof-of-concept, we develop a “stealth” adenoviral vector (sgCas9-Adv@TCM) for systemic cancer immunotherapy through dual blockades of PD-1/PD-L1 and TIGIT/CD155 pathways (Fig. 1a). First, the Adv vector that encodes spCas9 and PD-L1 guided RNA (sgCas9-Adv) is produced for PD-L1 genome editing as our previously described [11]. On the other hand, we construct a genetically engineered cell line to stably overexpress TIGIT for disruption of the TIGIT/CD155 immune inhibitory axis. At last, the sgCas9-Adv is coated with the TIGIT expressing cell membrane extracted from these bioengineered cells through a simple membrane extrusion process [19,20]. Our studies demonstrated that sgCas9-Adv@TCM can be effectively accumulated in the tumor site through intravenous injection, without causing severe side effects. We further validated the functions and performances of the as-prepared “stealth” adenoviral vector in the subcutaneous HCC model, as well as the orthotopic HCC model with spontaneous metastases in the lung, which elicited a robust antitumor response but with reduced antiviral immunity and irAEs. Building upon these data, we further demonstrate the clinical translation potential of sgCas9-Adv@TCM in clinically relevant PDO and PDX models established from resected HCC patients' tumor tissues. Overall, this study provides a highly translatable therapeutic intervention for HCC patients.

**Fig. 1:**
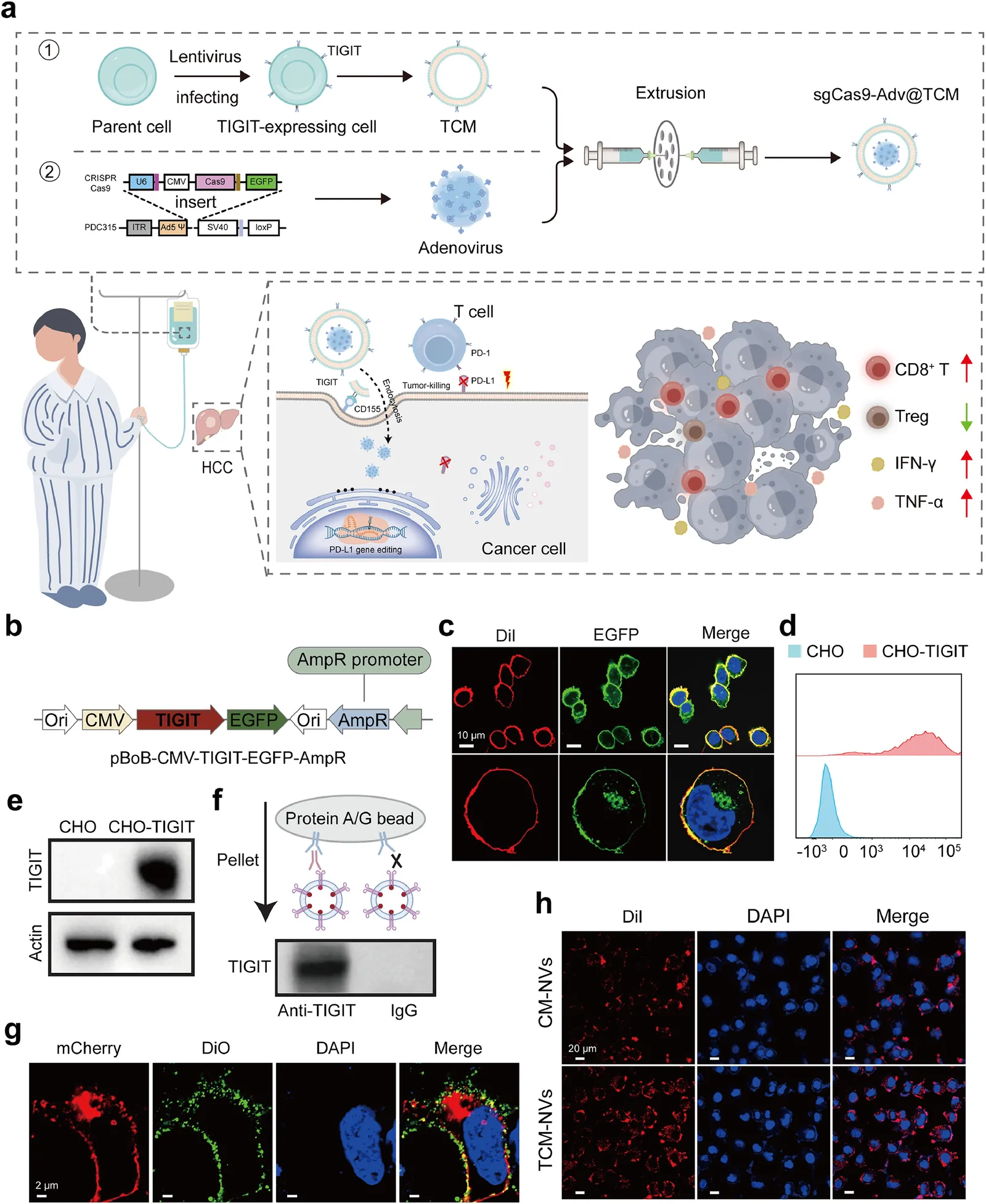
**Preparation and characterization of cell membranes expressing TIGIT protein (TCM) for camouflaging sgCas9-Adv.** (a) Schematic illustration of sgCas9-Adv@TCM for tumor immunotherapy via PD-L1 genome editing and TIGIT/CD155 blocking. (b) The design of the TIGIT-encoding plasmid to construct CHO-TIGIT cells. (c) Co-localization of TIGIT-EGFP with DiI-labeled cell membranes on CHO-TIGIT cells by CLSM. Scale bar: 10 µm. (d) The representative flow cytometric analysis of CHO-TIGIT cells by flow cytometry. (e) Verification of TIGIT expression in CHO-TIGIT cells by western blot. (f) Co-IP to examine the existence of TIGIT on the TCM-NVs. Anti-TIGIT antibody could pull down TCM-NVs, while IgG as a control could not bind to TIGIT proteins on TCM-NVs. (g) CLSM images to observe TCM-NVs binding to CD155-mCherry that is intentionally overexpressed on Hepa 1–6 cells. The colocalization of DiO-labeled TCM-NVs and PD-L1 proteins was represented by the yellow color. Scale bar: 2 µm. (h) CLSM images of Hepa1–6 cells incubated with DiI-labeled TCM-NVs for 4 h Scale bar: 20 µm.

## 2 Materials and methods

### 2.1 See the supporting information for experimental details

#### 2.2 Ethical approval

Human tumor specimens were acquired from patients undergoing surgical resection at Mengchao Hepatobiliary Hospital of Fujian Medical University. All enrolled patients were diagnosed with hepatocellular carcinoma and had not received any relevant treatment prior to surgery. Institutional review board—approved written and informed consent was obtained from patients with hepatocellular carcinoma to obtain tumor tissues for investigation. All studies were performed in accordance with ethical regulations and approved by the Ethics Review Committee of Mengchao Hepatobiliary Hospital of Fujian Medical University (KESHEN 2021_100_02). The Declaration of Helsinki guided all clinical investigations.

All animal procedures were reviewed and approved by the Animal Ethics Committee of the Mengchao Hepatobiliary Hospital of Fujian Medical University (MCHH-AEC-2022-08) in accordance with the “National Animal Management Regulations of China”.

## 3 Results

### 3.1 Preparation of cell membranes expressing TIGIT protein (TCM)

To prepare TCM, we established CHO cells stably expressing the mouse TIGIT protein (CHO-TIGIT) on the cell membrane by transfecting cells with lentiviral vectors (Fig. 1b and Fig. S1). In contrast to our previous studies and others that utilized parent cells such as HEK 293T or cancer cells [22,27], which pose risks of oncogenicity and undesirable immunogenicity, we opted for CHO cells. CHO cells are the most widely used hosts for producing recombinant biotherapeutic products and can efficiently integrate foreign genes with minimal immunogenicity [28]. Note that the EGFP protein tag was incorporated into the C-terminus of TIGIT, positioning it near the cell membrane’s inner leaflet, whereas TIGIT’s functional domain is extracellular. This design allows for direct observation of TIGIT expression through EGFP fluorescence without affecting TIGIT functions [29]. Confocal laser scanning microscopy (CLSM) observations showed excellent expression of TIGIT-EGFP on the plasma membrane of transfected cells, as indicated by co-localization with DiO cell membrane staining dyes (Fig. 1c). Flow cytometry showed almost 100% positive rate for TIGIT protein expression in stable cells (Fig. 1d and Fig. S2). The stable expression of the TIGIT proteins in CHO-TIGIT cells was also confirmed by western blot assays (Fig. 1e and Fig. S3). Collectively, these results demonstrate that the TIGIT proteins have been expressed successfully on CHO cell membranes.

TCM was then isolated from CHO-TIGIT cells by a combined process of disruption and multistep density gradient ultracentrifugation to remove the intracellular components [27]. Afterwards, the TCM was transformed into nanovesicles (TCM-NVs) through repeated sonication and extrusion with a polymeric membrane filter. The anti-TIGIT antibody was able to pull down TCM-NVs by immunoprecipitation (IP) assay, confirming their proper outside-out orientation for binding to CD155 (Fig. 1f and Fig. S4). The binding of TIGIT on immune cells to CD155 on cancer cells induces immune cell exhaustion and inhibits their anti-tumor efficacy [30]. To demonstrate whether TCM-NVs can disrupt the TIGIT/CD155 pathway by competitively occupying the binding sites of CD155 on cancer cells, CD155-mCherry expressing Hepa1–6 cells generated by transfection with CD155-mCherry plasmid, were treated with TCM-NVs at 4 °C for 1 h. As expected, a significant co-localization of DiO-labeled TCM-NVs and CD155-mCherry on Hepa1–6 cells was observed by CLSM (Fig. 1g). To further investigate the effect of TIGIT presentation on the internalization of NVs into cancer cells, Hepa1–6 cells were incubated with DiI-labeled NVs at 37 °C for 6 h, and CLSM images showed that TCM-NVs exhibited stronger internalization in Hepa1–6 cells compared to free NVs (CM-NVs) derived from the membranes of native CHO cells (Fig. 1h). Taken together, these results demonstrated that TCM-NVs can specifically interact with tumor cells via the interaction between TIGIT and CD155 and then enhance their internalization.

### 3.2 Synthesis and characterization of sgCas9-Adv@TCM

According to our recent report [11], sgCas9-Adv originated from an adenovirus serotype 5 (Ad5) vector was generated in HEK293A cells using Lipofectamine 3000 transfection reagent to co-transfect PD-L1 sgRNA/Cas9 pDC315 shuttle plasmid and pBHGlox (delta) E1, 3Cre backbone plasmid (Fig. 2a and Fig. S5). The sgCas9-Adv was purified, and its titers were determined by quantitative real-time polymerase chain reaction (qRT-PCR) after CsCl gradient centrifugation (Fig. S6). Then, the sgCas9-Adv was mixed with TCM and subjected to repeated sonication and extrusion with different-sized membrane filters to obtain the final product of sgCas9-Adv@TCM. Compared to naked sgCas9-Adv which showed a typical icosahedral capsid without envelope structure, the sgCas9-Adv@TCM visualized by transmission electron microscopy (TEM) after uranyl acetate staining had a distinct core-shell morphology, indicating successfully coating of TCM on sgCas9-Adv (Fig. 2b). In addition, dynamic light scattering (DLS) showed that the average hydrodynamic diameter of sgCas9-Adv slightly increased from 137.6 nm to 144.6 nm after coating with TCM, which was significantly larger than TCM vesicles (90.4 nm) (Fig. 2c). Meanwhile, the zeta potential of the uncoated sgCas9-Adv was −17.4 mV, which slightly decreased to −20.8 mV after encapsulation with the negatively charged cell membranes (−19.2 mV) (Fig 2d). An SDS-PAGE electrophoresis assay was also performed to determine whether the cell membrane successfully enveloped the viral particles. As shown in Fig. 2e, the electropherogram of sgCas9-Adv@TCM had the characteristic protein bands of both individual vesicles and viral particles. These physical characterizations confirmed the effective decoration of sgCas9-Adv with TCM for performing multiple functions.

**Fig. 2:**
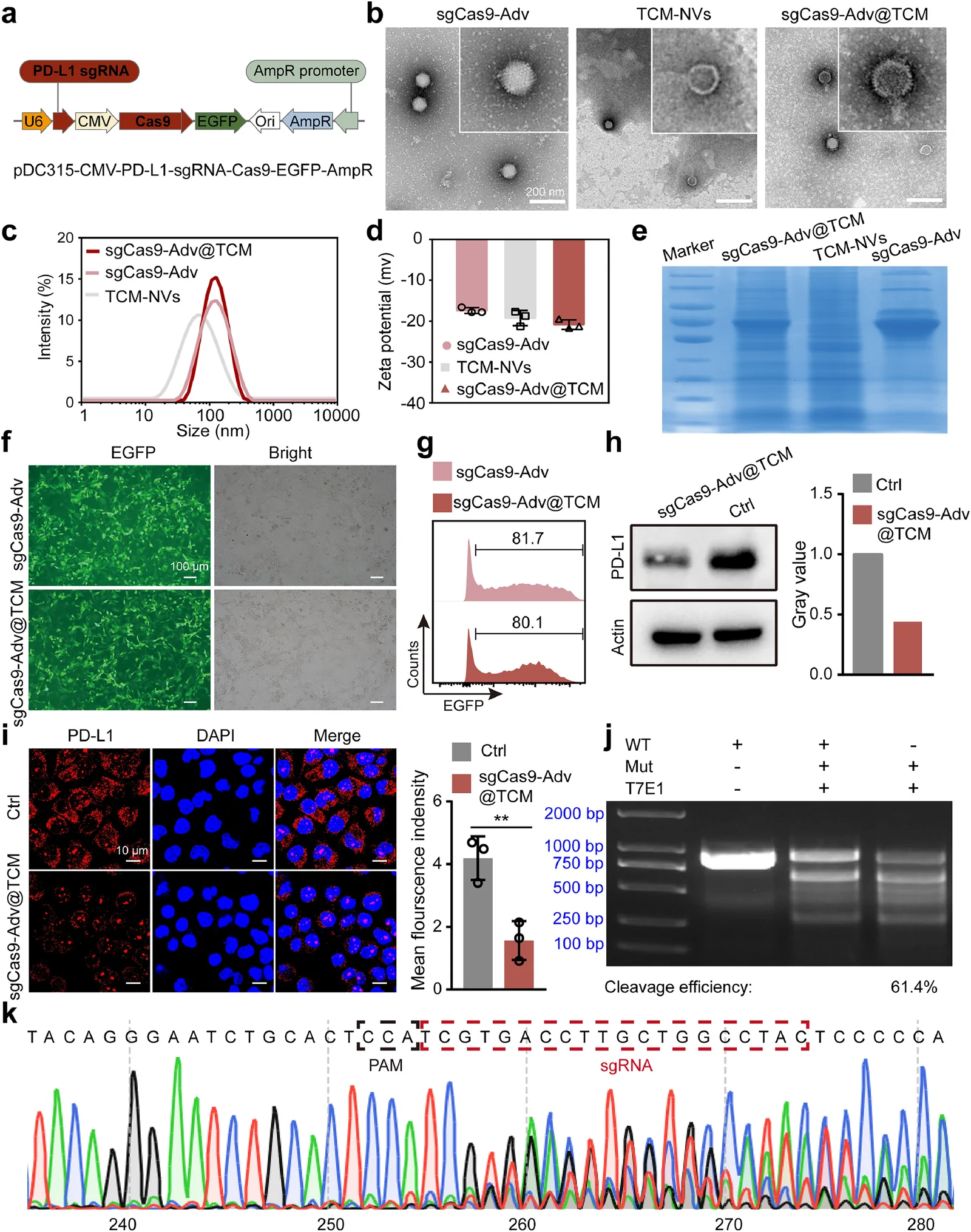
**Characterization of sgCas9-Adv@TCM in vitro.** (a) The design of the shuttle plasmids for the construction of sgCas9-Adv. (b) Transmission electron microscopy (TEM) images of sgCas9-Adv, TCM-NVs and sgCas9-Adv@TCM. (c, d) The size distribution (c) and zeta potential (d) of sgCas9-Adv, TCM-NVs and sgCas9-Adv@TCM as measured by DLS (*n* = 3). (e) SDS-PAGE protein analysis of sgCas9-Adv, TCM-NVs and sgCas9-Adv@TCM. (f, g) Infectivity of sgCas9-Adv@TCM on Hepa1–6 cells analyzed by fluorescence microscopy (f) and flow cytometry (g). (h) Western blot of PD-L1 protein expression of Hepa1–6 cells after infection with sgCas9-Adv@TCM. (i) Immunofluorescence image of PD-L1 protein expression of Hepa1–6 cells after infection with sgCas9-Adv@TCM and fluorescence intensity statistics from three fields. (j, k) The T7E1 assay (j) and Sanger sequencing (k) of PCR amplicon of the targeted PD-L1 locus in Hepa1–6 cells after transduction with sgCas9-Adv@TCM. Data in (d) and (i) are presented as mean ± SD. Statistical significance was calculated by two-tailed Student’s *t*-test in (i). ^⁎⁎^*P* < 0.01.

### 3.3 sgCas9-Adv@TCM mediated PD-L1 gene editing in vitro

Before investigating PD-L1 gene editing capability, we first examined the adenoviral vector-induced transgene expression in vitro. For this purpose, the sgCas9-Adv containing an enhanced green fluorescent protein (EGFP) cassette was incubated with Hepa1–6 cells. Fig. 2f, g and Fig. S7 verified that the sgCas9-Adv@TCM was comparable to that of uncoated sgCas9-Adv in terms of infecting Hepa1–6 cells, with over 80% EGFP-positive cells. This result indicates that membrane coating, along with sonication and extrusion processes, does not affect the activity of sgCas9-Adv.

Next, the gene editing efficiency of sgCas9-Adv@TCM was investigated by western blot and immunofluorescence imaging to detect the expression of PD-L1. On the basis of western blot analysis, the expression of PD-L1 was significantly downregulated in Hepa1–6 cells after infection with sgCas9-Adv@TCM for 48 h (Fig. 2h and Fig. S8). Additionally, the phenomenon was also replicable using immunofluorescence imaging (Fig. 2i). T7 endonuclease 1 (T7E1) digestion assay was used to further verify that disruption of PD-L1 expression actually occurred through genome editing, because T7E1 enzyme can recognize and cut incompletely matched DNA fragments created from indels (deletion or insertion) after CRISPR/Cas9-based genome editing [31]. PCR primers for this analysis were designed at 300 bp upstream and 700 bp downstream of the PD-L1 genomic regions. As depicted in Fig. 2j, cleaved bands at 300 and 700 bp were observed near the PD-L1 genomic locus, with an indel frequency of 61.4% determined by quantifying gray intensities of these bands relative to the uncut bands at 1000 bp, indicating the strong genomic disruption of PD-L1. Moreover, CRISPR-Cas9 transduction mediated by sgCas9-Adv@TCM showed two or more peaks around the protospacer adjacent motif (PAM) of PD-L1 loci by Sanger sequencing (Fig. 2k). Taken together, these experiments indicate that the sgCas9-Adv@TCM maintains PD-L1 gene editing ability after coating with TCM.

### 3.4 TCM mask sgCas9-Adv from the immune system for systemic delivery

Adenovirus-based vectors, especially Ad5, are highly immunogenic. After adenovirus infection, the body produces antibodies that can neutralize and clear the virus, protecting the body from adenovirus invasion [32]. Thus, a prerequisite for the systemic administration of sgCas9-Adv@TCM is that they should be resistant to pre-existing neutralizing antibodies (for Ad5-seropositive recipients) and avoid the generation of neutralizing antibodies (for Ad5-seronegative recipients). Firstly, to explore the role of the cell membrane in protecting the virus from neutralizing antibodies, a neutralization assay was carried out by monitoring EGFP transduction mediated by sgCas9-Adv@TCM in a culture medium supplemented with serum from the mice immunized with naked adenovirus (as a source of adenovirus-neutralizing antibodies). The workflow for this experimental procedure was depicted in Fig. 3a. Our observations revealed that the addition of immunized serum during co-incubation resulted in a significant inhibition of EGFP expression in cells treated with naked sgCas9-Adv, as observed in fluorescence microscope images. However, the fluorescence signal was still remarkably sustained in the sgCas9-Adv@TCM group, even at high titers of neutralizing antibodies, as shown in Fig. 3b. Consistently, flow cytometry revealed higher EGFP fluorescence in sgCas9-Adv@TCM-treated cells compared to naked sgCas9-Adv-treated cells in the immunized serum with the same titer of neutralizing antibodies (Fig. 3c and Fig. S9), further demonstrating the protective effect offered by the cell membrane towards neutralizing antibodies. Furthermore, our results revealed that wrapping the Adv vector into the cell membrane also led to a reduced generation of neutralizing antibodies in vivo (Fig. 3d).

**Fig. 3:**
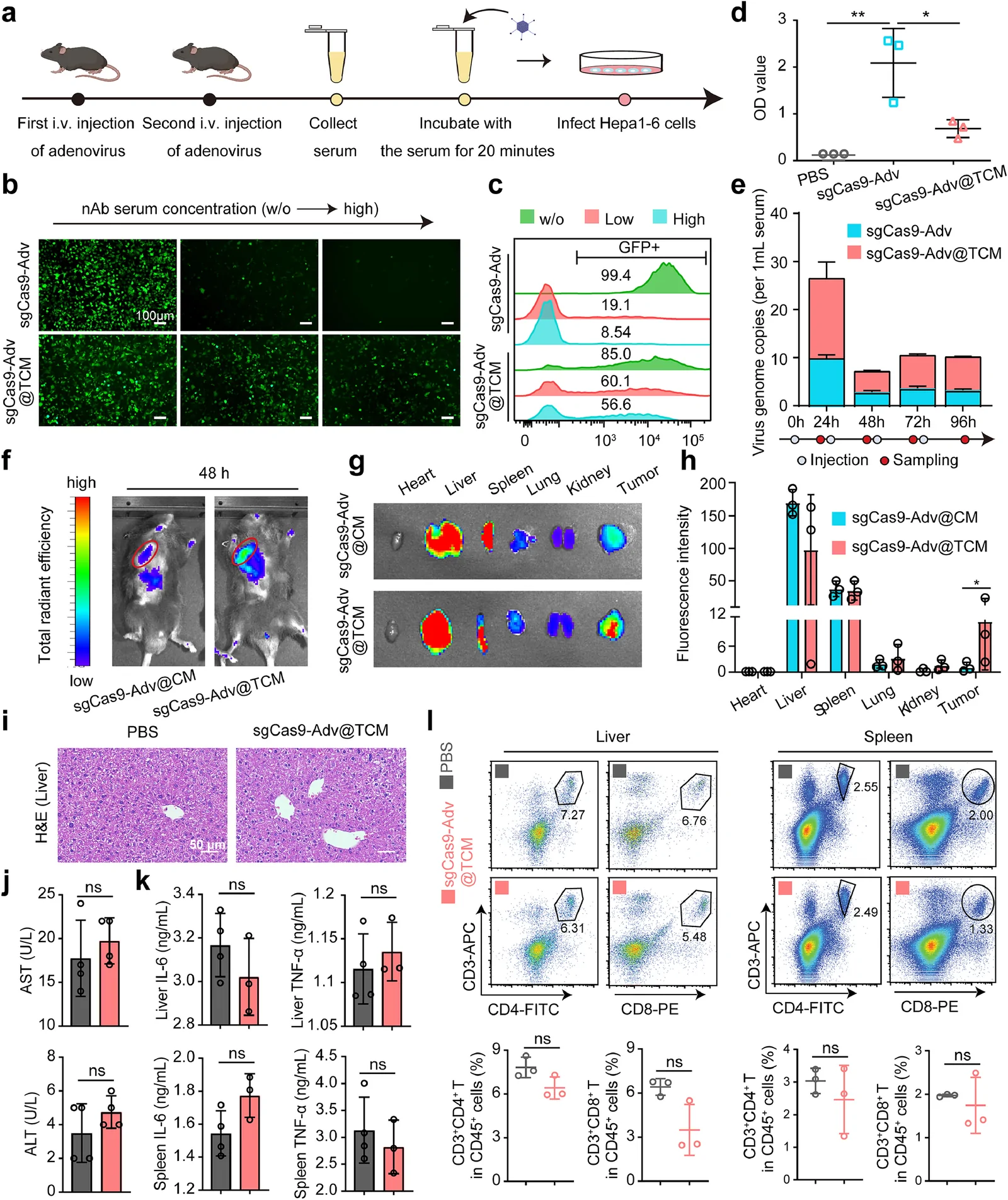
**Stealth effect of sgCas9-Adv@TCM in the host immune system.** (a) Schematic diagram of the experimental procedure for the resistance of sgCas9-Adv@TCM to neutralizing antibodies. Mice were intravenously injected with adenovirus to produce neutralizing antibodies in vivo, and the collected mouse serum with neutralizing antibodies was co-incubated with sgCas9-Adv or sgCas9-Adv@TCM prior to infection with Hepa1–6 cells. (b, c) Fluorescence micrographs (b) and flow cytometry results (c) of the infected Hepa1–6 cells. (d) ELISA detection of the neutralizing antibody level in serum after i.v. injection of sgCas9-Adv or sgCas9-Adv@TCM (*n* = 3). (e) Detection of adenovirus copy number in blood after repeated intravenous injection of sgCas9-Adv or sgCas9-Adv@TCM in mice. (f-h) In vivo fluorescence imaging of the mouse bearing Hepa1–6 tumor at 48 h after i.v. injection (f), ex vivo fluorescence imaging of major organs and tumor dissected from the mouse (g), and the mean radiant efficiency of tumor and major organs calculated by ImageJ software (h) (*n* = 3). (i-l) Safety evaluation of sgCas9-Adv@TCM from multiple aspects, including H&E staining of the liver (i), serum biochemical parameters (j), pro-inflammatory factors of IL-6 and TNF-α in liver and spleen (k) and immune T cell subsets in liver and spleen (l) (*n* = 3–4). Data are presented as mean ± SD. Statistical significance was calculated by one-way ANOVA with Tukey’s multiple comparison test. *P < 0.05, ^⁎⁎^*P* < 0.01. ns means no significant difference. Data in (d) to (l) are representative of two independent experiments.

In many cases, repeated injections are necessary to achieve satisfactory therapeutic efficacy against the aggressive malignant tumor. However, even in populations without pre-existing neutralizing antibodies against adenoviruses, Ad5-seronegative recipients would readily develop Ad5-seropositive status only after one Ad5-vector administration, and repeated treatments result in “accelerated clearance” of Ad5-based therapeutics [33]. To understand how the cell membrane envelope re-shapes the fate of such Adv in vivo, we utilized qRT-PCR to determine the genome copy numbers of adenovirus in the blood of mice at different time points after repeated intravenous injections. As shown in Fig. 3e, as the number of injections increased, the viral load detected in the blood decreased sharply. However, at any given time point, the viral load in the sgCas9-Adv@TCM group remained higher than that in the naked sgCas9-Adv group, suggesting that the TCM envelope can delay viral clearance by the host. In general, prolonged blood circulation is more conducive to the accumulation of nano-scaled particles, specifically viral particles, at tumor sites through the enhanced permeability and retention (EPR) effect [17,34]. Thus, coating viral surfaces with cell membranes enables effective delivery to tumor sites via systemic administration, with repeated dosing potentially achieving the desired therapeutic effect.

On the basis of these findings, we next evaluated whether cell membrane-coated Adv could accumulate in solid tumors in vivo. Mice with subcutaneous Hepa1–6 tumors were intravenously injected with DiR-labeled sgCas9-Adv@TCM, and whole-body fluorescence imaging was conducted at various time intervals to observe their distribution. Fig. 3f-h and Fig. S10 revealed that sgCas9-Adv@TCM had superior tumor accumulation at 48 h after injection compared to sgCas9-Adv@CM lacking TIGIT presentation, likely due to the binding between TIGIT receptor on sgCas9-Adv@TCM and CD155 on cancer cells to enhance tumor accumulation [22,35].

The safety of sgCas9-Adv@TCM was evaluated with particular regard to its impact on the liver and spleen, as significant accumulation of sgCas9-Adv@TCM was observed in these organs. After intravenous administration of sgCas9-Adv@TCM in healthy mice for 48 h, major organs and blood were collected to test hematoxylin and eosin (H&E) and serum biochemical parameters, respectively. Analysis of H&E-stained organs revealed no apparent signs of organ injury (Fig. 3i and Fig. S11). By measuring aspartate aminotransferase (AST) and alanine aminotransferase (ALT) in serum, the liver function of mice injected with sgCas9-Adv@TCM virus was shown to be unaffected (Fig. 3j and Fig. S12). Additionally, there was no upregulation of pro-inflammatory cytokines IL-6, TNF-α, IL-10, and IFN-γ in the liver and spleen after sgCas9-Adv@TCM treatment (Fig. 3k and Fig. S13), and the immune cell subpopulation status of CD4^+^ T and CD8^+^ T in the liver and spleen was also unchanged (Fig. 3l and Fig. S14). However, the naked virus exhibited apparent abnormalities in some of the aforementioned indexes. Wild-type Adv is known to be sequestered in Kupffer cells after intravenous administration to trigger hepatotoxicity [14]. To assess if this occurs with our cell membrane masked sgCas9-Adv@TCM, we further examined the toxicity to Kupffer cells. As shown in Fig. S15, sgCas9-Adv induced significant necrotic death of Kupffer cells in mice, whereas our sgCas9-Adv@CM did not show apparent toxicity, further demonstrating the safety of our cell membrane cloak strategy. These findings indicate that although sgCas9-Adv@TCM accumulated in the liver and spleen, it did not cause significant damage or an inflammatory risk.

### 3.5 Anti-tumor therapeutic effect of sgCas9-Adv@TCM on subcutaneous HCC model

Based on the aforementioned advantages, we subsequently established a subcutaneous Hepa1–6 mouse tumor model to evaluate the therapeutic effect of sgCas9-Adv@TCM after systemic administration (Fig. 4a). The mice with tumors that had grown to approximately 100 mm^3^ were administered with different formulations via intravenous injections, given once every 3 days for a total of 4 times. The formulations included PBS (G1), sgCas9-Adv (G2), sgCas9-Adv@CM (G3), physical mixture of sgCas9-Adv and TCM-NVs (sgCas9-Adv + TCM-NVs, G4), and sgCas9-Adv@TCM (G5) (Fig. 4b). As shown in Fig. 4c and d, there was no significant difference in tumor growth trend between the groups treated with sgCas9-Adv and PBS. This is because the naked virus can be rapidly cleared and inactivated by the host, making it difficult to accumulate at the tumor site to exert its anti-tumor effects despite carrying the CRISPR-Cas9 system for PD-L1 editing. After being enveloped by the cell membrane, the virus exhibited an enhanced anti-tumor effect. In particular, sgCas9-Adv coated with TCM (G5) significantly attenuated tumor growth by >80% comparing to the other groups including the cocktail of sgCas9-Adv and TCM-NVs (G4) at the equivalent dose after 14 days of treatment (Fig. 4e). Although >80% of the mice in other groups succumbed to tumor growth that exceeds the ethical limit within 28 days of treatment, all mice in the sgCas9-Adv@TCM group remained alive with minimal tumor burden (Fig. 4f, g and S16). Pathological observation of tumor slices from mice treated with different formulations using H&E, Ki67, and TUNEL staining revealed significant tumor damage in mice treated with the sgCas9-Adv@TCM (Fig. 4h and Fig. S17). Overall, these results demonstrate that the sgCas9-Adv@TCM with PD-L1 genome editing and TIGIT/CD155 blockade can suppress tumor growth through systemic delivery.

**Fig. 4:**
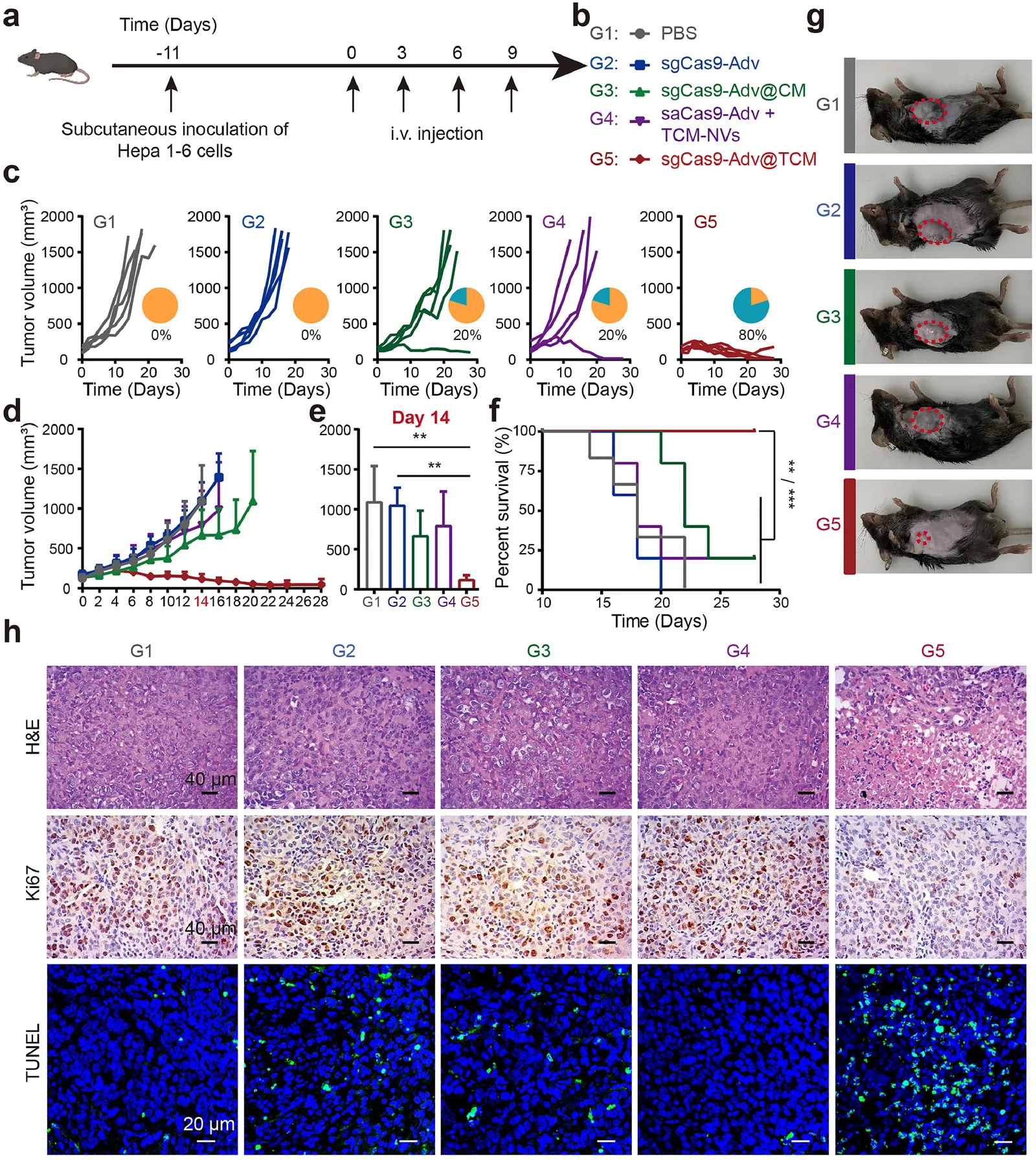
**Therapeutic efficacy of sgCas9-Adv@TCM on Hepa1–6 subcutaneous tumors.** (a) Schematic illustration of the setting of Hepa 1–6 subcutaneous tumor model to receive different treatments. (b) Group information of different treatments. (c, d) individual tumor growth kinetics (c) and average tumor volume curves (d) in different groups. Growth curves were stopped when the tumor volume of the corresponding group exceeded 1500 mm^3^. (e, f) Tumor volume on the 14th day (e) and Kaplan–Meier survival curves (f) after receiving different treatments as indicated (*n* = 5). (g) Representative photograph of Hepa1–6 tumor-bearing mice after receiving different treatments as indicated. The tumor area was labeled with a red circle. (h) H&E, Ki67 and TUNEL staining of tumor slices for different treatments. Data in (d) and (e) are presented as mean ± SD. Statistical significance was calculated by one-way ANOVA with Tukey’s multiple comparison test in (e) and log-rank (Mantel-Cox) test in (f). ^⁎⁎^*P* < 0.01, ^⁎⁎⁎^*P* < 0.001. Data in (b) to (h) are representative of two independent experiments.

Since our sgCas9-Adv@TCM performs its functions through double blocking of PD-L1 and TIGIT/CD155 signaling, we wonder whether it could also treat CD155-negative tumors. Therefore, we further evaluated the antitumor efficacy of sgCas9-Adv@TCM in a subcutaneous CD155-KO HCC model. The results in Fig. S18 demonstrate its effectiveness against CD155-negative tumors. Although the knockout of CD155 reduces the uptake of sgCas9-Adv@TCM at the tumor site, CD155 also functions as an immune checkpoint molecule by binding to TIGIT on T cells for promoting T cell exhaustion, which might limit the therapeutic efficacy. This contradictory role of CD155 allows sgCas9-Adv@TCM to effectively exert antitumor effects in CD155-negative tumors via PD-L1 blockade, highlighting its broad therapeutic potential.

### 3.6 sgCas9-Adv@TCM elicited anti-tumor immunity

Next, we aimed to analyze the cellular and molecular mechanisms underlying the robust antitumor effects of sgCas9-Adv@TCM. Firstly, we examined whether sgCas9-Adv@TCM has the ability to transduce cancer cells in vivo after systemic delivery, by checking the expression of EGFP (which was inserted in the same adenoviral genome together with the sgRNA and Cas9) on frozen slices of tumor tissues. Strikingly, sgCas9-Adv@TCM showed the highest level of transduction efficiency at the tumor sites compared to the other groups (Fig. 5a), which was attributed to the favorable tumor accumulation and activity facilitated by TCM coating. Concomitantly, immunohistochemistry and western blotting analysis revealed a significant downregulation of PD-L1 protein expression through sgCas9-Adv@TCM-mediated genome editing (Fig. 5b, c and Fig. S19). To further delineate antitumor response elicited by sgCas9-Adv@TCM, we performed flow cytometry-based immune cell subset analysis in the tumor tissue. The most enrichment of fluorescent signals of CD4^+^ *T* cells and CD8^+^ T cells was observed in the sgCas9-Adv@TCM group compared to other treated groups. Meanwhile, sgCas9-Adv@TCM remarkably enhanced the repertoires of IFN-γ^+^CD8^+^ T cells while reducing the infiltration of immunosuppressive Treg cells in tumor tissues (Fig. 5d and Fig. S20). Meaningfully, there were much fewer PD-1^+^TIM-3^+^ exhaustion phenotype cells among tumor infiltrating CD8^+^ T cells in sgCas9-Adv@TCM-treated mice (Fig. S21). Furthermore, we analyzed the cytokines in the tumor by ELISA, and the result showed that the sgCas9-Adv@TCM significantly increased the immunostimulatory cytokines (TNF-α and IFN-γ) (Fig. S22). The tumor sections stained with immunofluorescence also confirmed that tumor-infiltrating CD4^+^ T cells and CD8^+^ T cells were most abundant after treating with sgCas9-Adv@TCM (Fig. 5e). These results collectively indicate that our designed adenoviral vector, which integrates anti-viral immune evasion, PD-L1 gene editing, and CD155/TIGIT blockade functions, can effectively modulate the immunosuppressive tumor microenvironment to strengthen antitumor immunity.

**Fig. 5:**
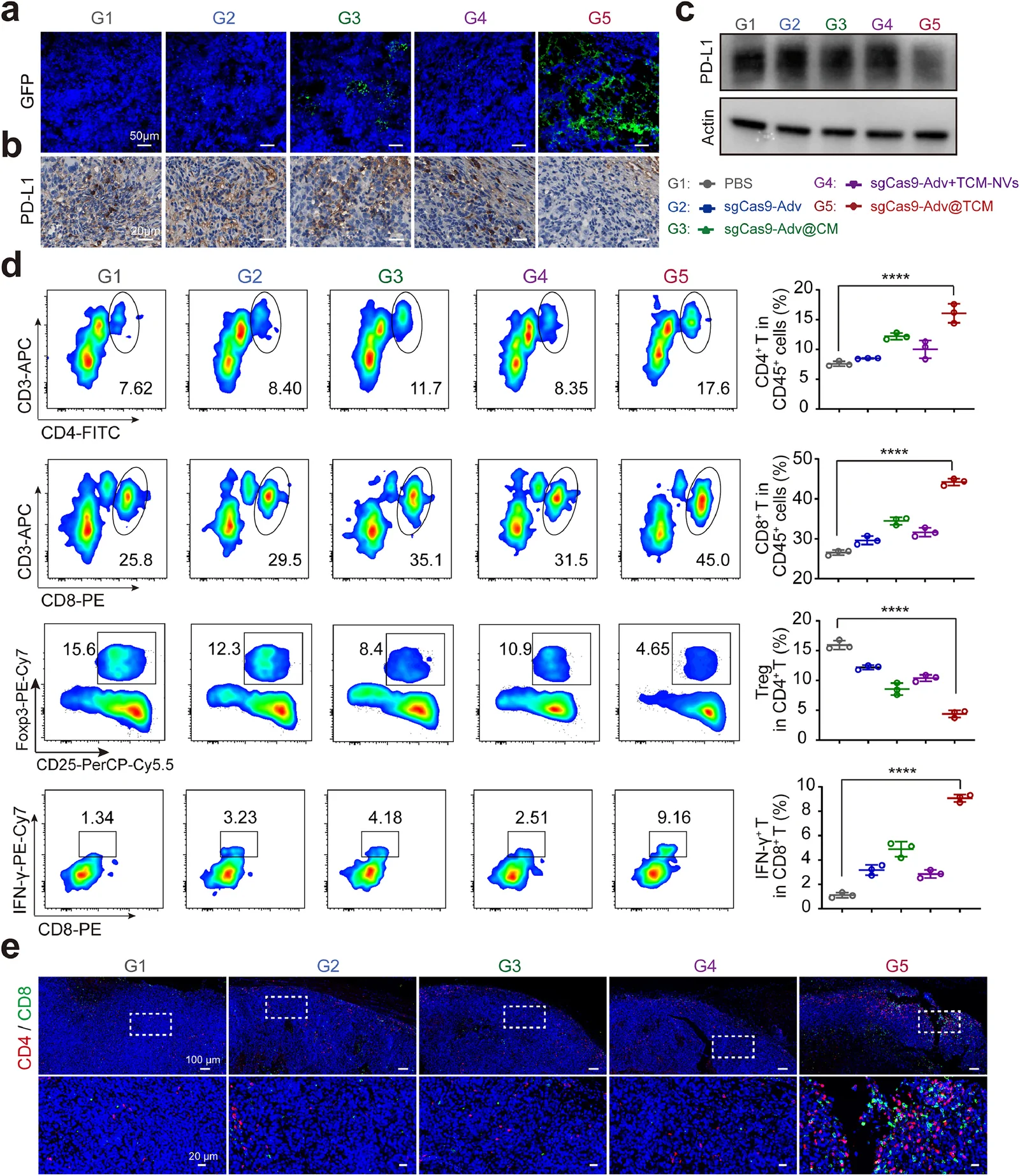
**Evaluation of the intra-tumoral immune response.** (a, b) EGFP fluorescence (a) and IHC depicting PD-L1 (b) of tumor slices for different treatments. (c) Western blot analysis of PD-L1 expression in the tumors with indicated treatments. (d) The percentage of tumor-infiltration of CD3^+^CD4^+^ T, CD3^+^CD8^+^ T, Treg and IFN-γ^+^CD8^+^ T cells after receiving different treatments, which were analyzed by flow cytometry on the 4th day (*n* = 4). (e) Immunofluorescence staining of tumor-infiltrating CD4^+^*T* cells (red) and CD8^+^ T cells (green) after receiving different treatments. Data in (d) are presented as mean ± SD. Statistical significance was calculated by one-way ANOVA with Tukey’s multiple comparison test in (d). ^⁎⁎⁎⁎^*P* < 0.0001. Data representative of two independent experiments.

To further evaluate the role of T cell populations in the antitumor immune response, additional experiments were performed to examine the effects of sgCas9-Adv@TCM on tumor growth in mice after depleting CD8^+^ or CD4^+^ T cells. As shown in Fig. S23, depletion of either CD8^+^ or CD4^+^ T cells using anti-CD8 or anti-CD4 antibodies attenuated the effectiveness of sgCas9-Adv@TCM, with a more pronounced impact observed for anti-CD8 antibody treatment. These findings collectively demonstrate that sgCas9-Adv@TCM primarily reinvigorates CD8^+^ T cells through the dual blockade of PD-1/PD-L1 and TIGIT/CD155 pathways, while CD4^+^ T cells also play a coordinated role in orchestrating the antitumor immune response.

### 3.7 Anti-tumor therapeutic effect of sgCas9-Adv@TCM on orthotopic HCC model

To determine the translatability of our designed adenoviral vector, we further explored and compared the efficacy of sgCas9-Adv@TCM with commercially available ICB monoclonal antibodies in an orthotopic HCC mouse model that more closely mimics the microenvironment in which tumor cells grow in the liver. Hepa1–6 cells that expressed firefly luciferase (Hepa1–6-Luc) were surgically inoculated into the liver lobes of C57BL/6 mice on day 0, and sgCas9-Adv@TCM or therapeutic antibodies were injected four times on the day of 8, 11, 14 and 17, respectively (Fig. 6a). Bioluminescence imaging was used to track the tumor burdens every 7 days from days 7 to 98. The results of Fig. 6b, c and Fig. S24, S25 demonstrated that mice treated with sgCas9-Adv@TCM significantly retarded tumor growth comparing to mice treated with PBS which experienced rapid tumor growth. Although effective in delaying tumor growth, neither a single anti-PD-L1 nor a single anti-TIGIT antibody could induce tumor regression, with only one of six mice experiencing complete tumor regression in both groups (1/6, 16.7%). When Anti-PD-L1 and Anti-TIGIT were administered in combination (Anti-PD-L1+Anti-TIGIT group), the rate of complete tumor regression increased to 50.0% (3/6), but still, the remaining two out of three mice exhibited rapid tumor growth on the 91st day during observation. In contrast, mice treated with sgCas9-Adv@TCM exhibited the most distinct response, with response rate significantly improved to 66.7% (4/6), resulting in complete regression of the majority of tumors; meanwhile, it also showed significant suppressive effects on the tumors that were not cured. Moreover, all mice treated with sgCas9-Adv@TCM remained alive throughout the study (Fig. 6d). The treatment effect was further validated through photographs of livers as well as H&E staining images for their sections. Systemic administration of sgCas9-Adv@TCM was not only effective in suppressing primary tumors but also showed significant prevention of metastasis in lung tissues, as evidenced by Fig. 6e and f. These results suggest that the sgCas9-Adv@TCM therapeutic regimen shows tremendous promise to enhance the efficacy of standard of care checkpoint blockade therapy in advanced HCC to eradicate primary tumors and spontaneous metastases.

**Fig. 6:**
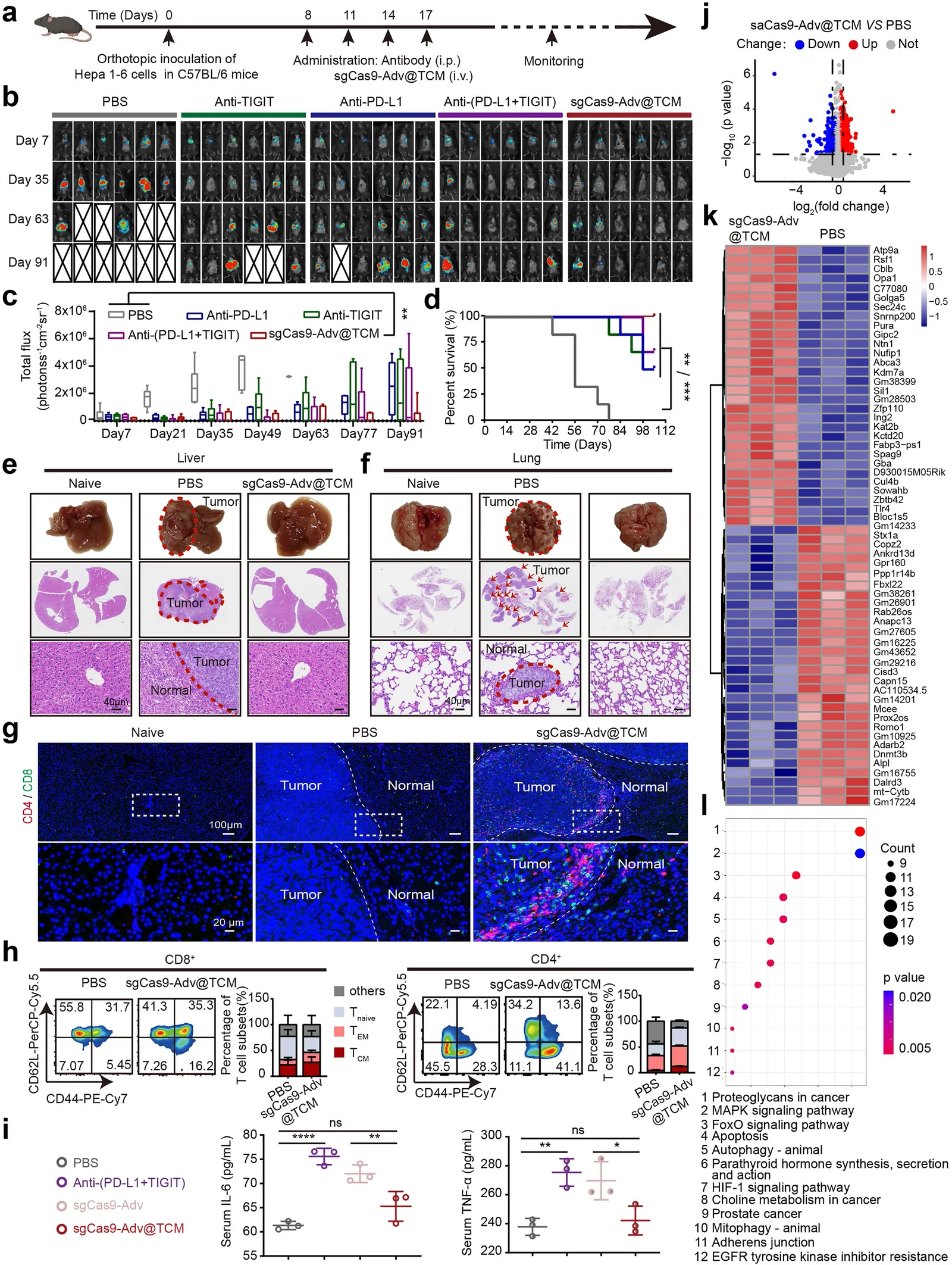
**Therapeutic efficacy of sgCas9-Adv@TCM on Hepa1–6 orthotopic tumors.** (a) Schematic illustration of setting the Hepa1–6 orthotopic tumor model to receive different treatments. (b) Bioluminescence imaging of mice in each group every one week as indicated. (c) Average tumor growth kinetics in different groups, as calculated from total flux in each mouse (*n* = 6). (d) The survival curves of orthotopic Hepa1–6 tumor-bearing mice after receiving different treatments as indicated (*n* = 6). (e, f) Representative images and H&E staining of the liver and lung of mice in each group on day 105 after receiving different treatments. (g) Immunofluorescence staining of tumor-infiltrating CD4^+^*T* cells (red) and CD8^+^ T cells (green) on the day 105 after receiving different treatments. (h) The percentage of central memory T cells (CD4^+^CD44^high^CD62L^high^ and CD8^+^CD44^high^CD62L^high^) and effective memory T cells (CD4^+^CD44^high^CD62L^low^ and CD8^+^CD44^high^CD62L^low^) in the spleen after receiving different treatments, which were analyzed by flow cytometry on the 35th day. Data are presented as mean ± SD (*n* = 4). (i) The levels of IL-6 and TNF-α in peripheral blood after receiving different treatments. (j-l) Transcriptomic analysis of Hepa1–6 orthotopic tumors after the treatment of sgCas9-Adv@TCM, including volcano plot, heatmap of DEGs and KEGG enrichment analysis. Data in (c), (h) and (i) are presented as mean ± SD. Statistical significance was calculated by one-way ANOVA with Tukey’s multiple comparison test in (c, i) and log-rank (Mantel-Cox) test in (d). *P < 0.05, ^⁎⁎^*P* < 0.01, ^⁎⁎⁎^*P* < 0.001, ^⁎⁎⁎⁎^*P* < 0.0001. ns means no significant difference. Data in (b) to (i) is representative of two independent experiments.

Moreover, immunofluorescence images of liver sections revealed that, in contrast to PBS-treated mice where effector T cells were sparsely present in both normal liver and cancerous liver tissue, sgCas9-Adv@TCM-treated mice exhibited a substantial infiltration of CD4^+^ and CD8^+^ T cells surrounding the cancerous tissue, with some T cells even penetrating into deep tumor area (Fig. 6 and Fig. S26). Further analysis of the activation state of CD8+ T cells within the tumors revealed a significant elevation in CD69 expression, a well-established marker of T cell activation, in the sgCas9-Adv@TCM-treated group (Fig. S27).

Furthermore, we assessed memory T cells in the spleen and observed a significant increase in the ratio of both central memory T cells (CD4^+^CD44^high^CD62L^high^ and CD8^+^CD44^high^CD62L^high)^ and effector memory T cells (CD4^+^CD44^high^CD62L^low^ and CD8^+^CD44^high^CD62L^low^) in the sgCas9-Adv@TCM group compared to the PBS group (Fig. 6h and Fig. S28). These findings suggest that the sgCas9-Adv@TCM treatment can induce a persistent memory immune response, which contributes to preventing tumor relapse or metastasis. Meanwhile, we noticed that naked sgCas9-Adv and combinational Anti-PD-L1+Anti-TIGIT treatments significantly increased the levels of IL-6, TNF-α, IFN-γ, and IL-10 in the mice, while sgCas9-Adv@TCM did not cause these irAEs that are associated with cytokine release syndrome (CRS) or systemic inflammation (Fig. 6i and Fig. S29) [36,37].

In order to gain more insights into the therapeutic efficacy of sgCas9-Adv@TCM, we performed bulk RNA sequencing on the tumor tissues. The volcano plot in Fig. 6j showed that the sgCas9-Adv@TCM treatment resulted in downregulation of 451 transcripts and upregulation of 1683 transcripts compared to PBS treatment. Further analysis of these differentially regulated genes using Kyoto Encyclopedia of Genes and Genomes (KEGG) suggested they were mostly enriched in pathways involving apoptosis, autophagy, and tumor metabolism (Fig. 6k and l). These results underscore the immune response elicited by sgCas9-Adv@TCM to effectively eliminate cancer cells from multiple aspects.

### 3.8 Preparation of humanized sgCas9-Adv@TCM

To advance the clinical translation of our Adv-functionalized design for human applications, we next created a humanized version of sgCas9-Adv@TCM. This involved substituting the sgRNA originally targeting mouse PD-L1 with the one targeting the human counterpart. Additionally, we replaced the TCM with that sourced from human 293A cells, which were engineered to express human TIGIT stably. After purifying and determining the viral titers via qRT-PCR, we compared the transgene expression of different humanized Adv constructs in human liver cancer cell lines, such as SK-Hep and SMMC-7721 cells. Compared to viruses lacking sgRNA (Cas9-Adv), viruses containing two different sgRNAs targeting to different loci of the PD-L1 gene (sg-1 Cas9-Adv and sg-2 Cas9-Adv) are equivalent in infection efficiency, as evidenced by the similar EGFP expression in the cells across all groups after infection with viruses at a multiplicity of infection (MOI) of 100 for 24h (Fig. S30a, S30b). Both sg-1 Cas9-Adv and sg-2 Cas9-Adv efficiently downregulated PD-L1 expression of SK-Hep and SMMC-7721 cells (Fig. S30c), and we only selected sg-2 Cas9-Adv for the following experiments. Accordingly, significant indel mutations were identified at genomic loci of PD-L1. This is exemplified by observations on SMMC-7721 cells infected with sg-2 Cas9-Adv through T7E1 assay (Fig. S30d) and Sanger sequencing (Fig. S30e). On the other hand, we generated human TIGIT protein in 293A cells using lentiviral transduction (Fig. S30f). Flow cytometry (Fig. S30g) and Western blot analyses (Fig. S30h) validated the successful display of human TIGIT protein on 293A cells. Finally, we isolated cell membranes from the TIGIT-expressing 293A cells and coated them onto sgCas9-Adv to engineer the humanized sgCas9-Adv@TCM, following the protocols utilized for the murine sgCas9-Adv@TCM as outlined previously.

### 3.9 Preclinical validation of humanized sgCas9-Adv@TCM on PDO and PDX models

PDO and PDX models were derived from tumor tissues that were acquired during surgical resection from two patients diagnosed with HCC (Table S1). The HCC organoid established from patient I (Fig. 7a) was found to preserve the histological properties of the originating primary tumors, as evidenced by hematoxylin and eosin (H&E) staining. Additional immunohistochemical analysis, focusing on the characteristic biomarkers of HCC, underscored the fidelity of the organoids in recapitulating the original tissue's expression of alpha-fetoprotein (AFP) and Glypican 3 (GPC3). Thus, the findings presented in Fig. 7b eloquently affirm that the HCC organoids faithfully retain the phenotypic characteristics of their originating tumors. Subsequently, a HCC organoid-T cell co-culture system was crafted to ascertain the efficacy of sgCas9-AdV@TCM as a potential CICB inhibitor (Fig. 7a), aimed at revitalizing the antitumor effector functions of T cells. For this purpose, HCC organoids were infected with an adenoviral vector for 24h. Fluorescence microscope images revealed that, irrespective of the insertion of sgRNA or surface coating with cell membranes, viral vectors demonstrate remarkably high infectivity towards PDOs, as nearly all PDOs exhibit vibrant EGFP green fluorescence (Fig. 7c and Fig. S31). Meanwhile, the PD-L1 expression level decreased when PDOs were incubated with sgCas9-AdV or sgCas9-AdV@TCM (Fig. 7d and Fig. S32). We further incubated these adenovirus-infected PDOs with CD8^+^ T cells, derived from autologous PBMCs and stimulated with anti-CD3/anti-CD28 and IL-2. Compared with other groups, caspase3/7 staining results indicate that cells intervened by adenoviral vectors with PD-L1 gene editing capability are more susceptible to CD8^+^ T cell attack, exhibiting more pronounced apoptosis (Fig. 7e and Fig. S33). Furthermore, despite the comparable infectivity between the sgCas9-AdV and sgCas9-AdV@TCM groups, the higher PDO apoptosis observed in the sgCas9-AdV@TCM group may be ascribed to the presence of TIGIT protein on TCM to further block CD155.

**Fig. 7:**
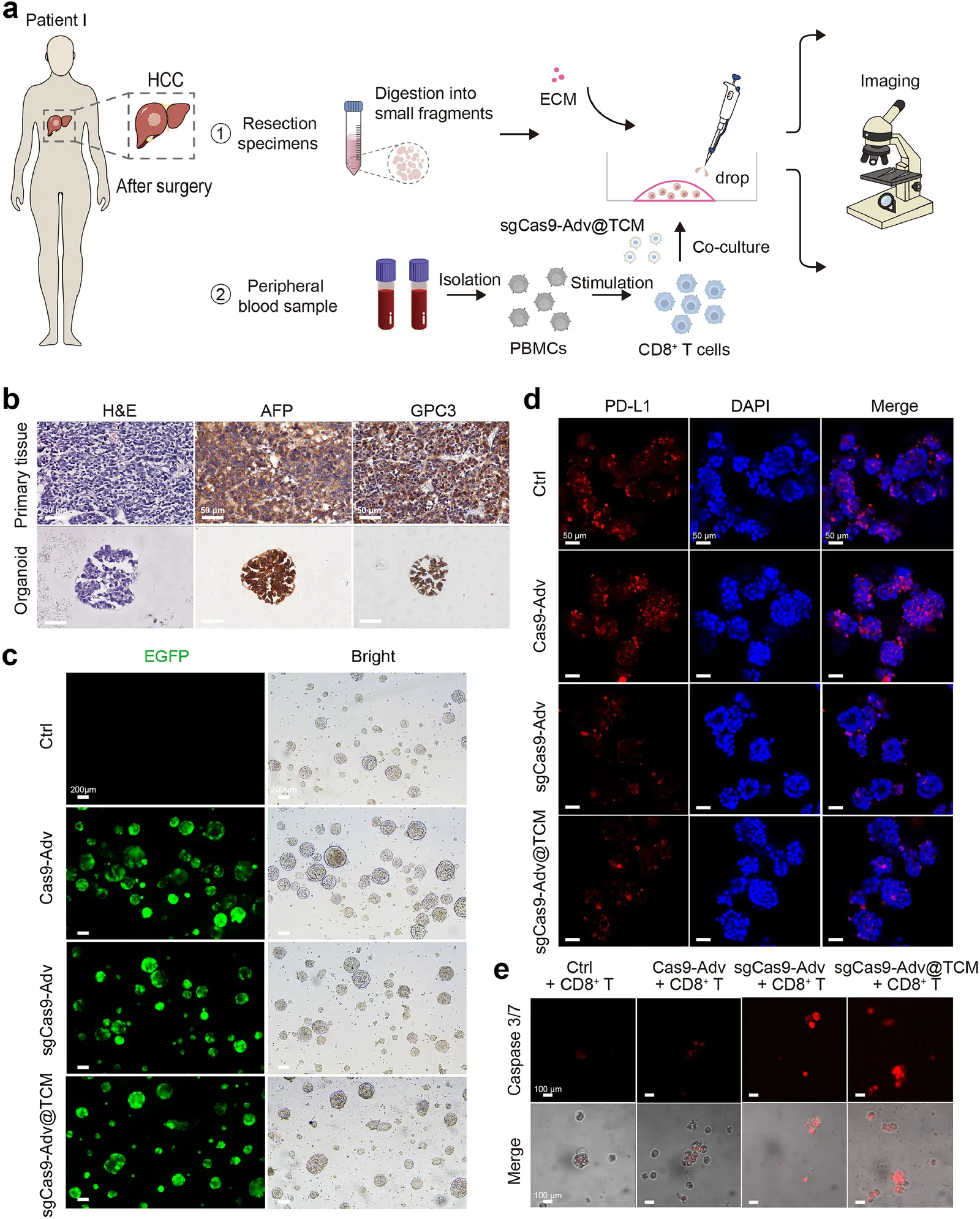
**Therapeutic efficacy of sgCas9-Adv@TCM on PDO model.** (a) Schematic illustration of the preparation and treatment of PDOs. (b) Histological sections of primary HCC tissues and PDOs stained with H&E. The HCC marker AFP and GPC3 were detected by immunohistochemistry. Scale bar: 50 µm. (c) Infectivity of Cas9-Adv, sgCas9Adv and sgCas9-Adv@TCM on PDOs analyzed by fluorescence microscopy. Scale bar: 200 µm (d) Immunofluorescence of PD-L1 protein expression of PDOs after infection with Cas9-Adv, sgCas9-Adv and sgCas9-Adv@TCM. Scale bar: 50 µm. (e) The CLSM images of PDOs stained with the caspase 3/7 probe after different treatments. Scale bar: 100 µm.

To further elucidate the therapeutic potential of humanized sgCas9-Adv@TCM in clinically relevant animal models, we established a hepatocellular carcinoma (HCC) patient-derived xenograft (PDX) model in NCG mice. A primary tumor specimen extracted from patient II was meticulously sectioned into small pieces and subsequently engrafted into the axilla of NCG mice, undergoing three passages (Fig. 8a). Following the confirmation of the successful establishment of the PDX model through histological examination using hematoxylin and eosin (H&E) staining and immunohistochemistry staining for AFP/GPC3/CD155/PD-L1 (Fig. 8b and Fig. S34), intratumoral injections of sgCas9-Adv@TCM were administered to the mice on days 0 and 4. Concurrently, autologous pre-stimulated CD8^+^ T cells were intravenously infused into the mice on days 2 and 6 (Fig. 8a). No obvious change in body weight was observed during the whole treatment course (Fig. 8c). In comparison to the control group (G1, only treated with PBS), solely relying on CD8^+^ T cell infusion could only transiently suppress tumor growth in the early stages (G2, PBS+ T). Even post-intervention with Anti-PD-L1+Anti-TIGIT (G3), the cytotoxic killing of T cells on the tumor remained insignificantly altered. Following a 10-day treatment regimen, the vast majority of PDX tumors among these three treatment cohorts reached a volume of about 1500 mm^3^. However, the incorporation of sgCas9-Adv@TCM with CD8^+^ T cell infusion yielded the slowest tumor growth rate, with the mean tumor size in this group measuring <500 mm^3^ (Fig. 8d and e). The better therapeutic outcome of sgCas9-Adv@TCM was further corroborated by the tumor photographs and weights recorded on day 10 (Fig. 8f and g). To clarify the underlying mechanism of anti-tumor effects, we further analyzed the infiltration level and activation status of T cells in the tumor through flow cytometry (Fig. 8h and Fig. S35). The results showed that intravenously infused T cells could effectively accumulate in the tumor, and the infiltration levels were comparable among different treatment groups. However, in the sgCas9-Adv@TCM -treated group, a higher proportion of T cells were found to secrete effector cytokines such as IFN-γ and granzyme B, indicating that our designed virus vector can increase the anti-tumor activity of T cells inside the tumor through immune checkpoint blocking. Thus, these findings indicate that our sgCas9-Adv@TCM could effectively suppress PDX growth in vivo by activating the antitumor immunity.

**Fig. 8:**
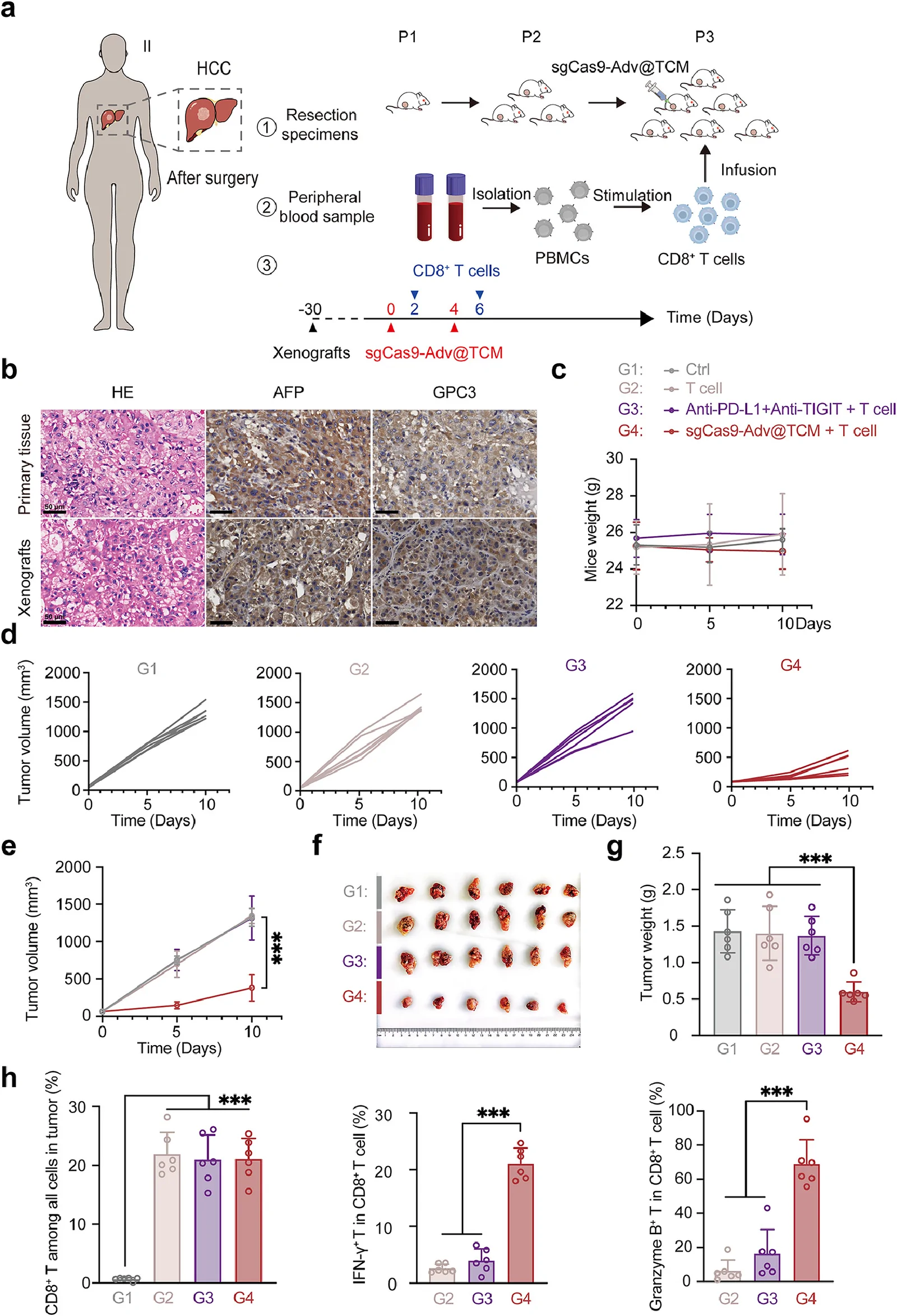
**Therapeutic efficacy of sgCas9-Adv@TCM on PDX model.** (a) Schematic illustration of the preparation and treatment of HCC patient-derived PDX. (b) Histological sections of primary HCC tissues and PDOs stained with H&E. The HCC marker AFP and GPC3 were detected by immunohistochemistry. Scale bar: 50 µm. (c) The body weight of mice after various treatments. (d, e) individual tumor growth kinetics (d) and average tumor volume curves (e) in different groups. (f, g) The tumor photographs (f) and weight (g) of each group of the PDX model at the end of treatment. (h) The percentage of tumor-infiltration of CD8^+^ T, IFN-γ^+^CD8^+^ T and Granzyme B^+^CD8^+^ T cells after receiving different treatments (*n* = 6), which were analyzed by flow cytometry. Data in (c), (e), (g) and (h) are presented as mean ± SD. Statistical significance was calculated by one-way ANOVA with Tukey’s multiple comparison test in (e, g, h). ^⁎⁎⁎^*P* < 0.001. ns means no significant difference.

## 4 Discussion and conclusion

Although immune checkpoint blockades (ICBs) targeting the CTLA-4 and PD-1/PD-L1 pathways have shown promising results in cancer treatment, they also encounter challenges such as low response rates and adaptive drug resistance [4]. To further increase response rates, academic and pharmaceutical studies are actively focusing on the development of alternative checkpoint therapies that can either complement or substitute anti-PD1 and anti-CTLA4 treatments [6]. In this context, treatment with combined immune checkpoint blockade (CICB) that simultaneously blocks the PD-1/PD-L1 and TIGIT/CD155 coinhibitory receptor pathways demonstrated favorable survival outcomes in both mice and humans [38,39]. However, the primary endpoint was not met in the Phase II trials when vibostolimab (anti-TIGIT) and perbrolizumab (anti-PD-1) were co-formulated, according to the latest clinical data in non-small cell lung cancer (NSCLC) [26]. Although the causes behind the unexpectedly negative findings in these studies are not yet fully understood at the preclinical and clinical levels, the transient work manner and cumulative immune-related adverse events (irAEs) may limit the application of the monoclonal antibody-based ICB agents. The main objective of this study was to create a CICB strategy for targeting both the PD-1/PD-L1 and TIGIT/CD155 pathways without relying on antibodies, while ensuring safety and controllability for potential clinical use.

For this purpose, we used our recently reported adenoviral vector-based CRISPR-Cas9 gene editing tool (sgCas9-Adv) to block PD-1/PD-L1 axis, which is distinct from clinical PD-1/PD-L1 checkpoint inhibitors, and it achieves permanent silence of PD-L1 at the genomic level. Additionally, the surface of sgCas9-Adv was adorned with a genetically engineered cell membrane that displays TIGIT proteins (TCM), serving as a competitive blockade for the TIGIT/CD155 axis. They emulate the natural interaction between TIGIT on immune cells and CD155 on cancer cells, potentially reducing the incidence of irAEs compared to clinical TIGIT/CD155 checkpoint inhibitors. In fact, several studies, including our own, have reported that genetically engineered cell membrane expression of immune signaling molecules, such as PD-1 protein, has lower immune-related adverse effects than anti-PD-L1, while maintaining comparable therapeutic efficacy [22,29,40]. More importantly, the cell membrane's ability to stealthily shield adenoviral vectors (Adv) from the host immune system presents a potential unprecedented opportunity for systemic administration, as opposed to locoregional administration, in the treatment of refractory tumors at late stage [19,20].

In proof-of-principle experiments, the sgCas9-Adv cloaked with TCM substantially resisted inactivation by humoral factors and avoided exuberant activation of innate immunity compared with naked sgCas9-Adv, allowing systemic and repeated administration for cancer treatment with minimized risks. To prioritize biological safety, we deliberately chose CHO cells (a commonly used cell line for production) to isolate cell membrane for Adv decoration, as opposed to previously reported cell membrane isolation from immortalized cell lines (such as cancer cells [19] or HEK 293 cells [20]) which may carry a risk of tumorigenicity. As a result, the sgCas9-Adv@TCM was proved to suppress tumor growth and prolong the survival of mice with subcutaneous and orthotopic HCC models, which was even superior to the standard CICB regimen of the combined anti-PD-L1 and anti-TIGIT. Importantly, sgCas9-Adv@TCM showed significantly lower risks of irAEs, as evidenced by much lower levels of inflammatory cytokines such as IL-6 and TNF-α in serum after treatment.

In the PDX model, we originally intended to administer sgCas9-Adv@TCM into mice intravenously. However, despite the mitigation of viral immunogenicity through cell membrane camouflage, systemic delivery of the virus swiftly resulted in mortality among severely immunocompromised NCG mice. Consequently, as a proof of concept, we chose to employ intratumoral (i.t.) administration of the virus to attenuate its systemic repercussions. In forthcoming investigations, the utilization of alternative humanized mouse models such as huHSC—NCG, coupled with hematopoietic stem cell (CD34^+^HSC) transplantation to reconstitute the human immune system, holds great promise for alleviating or diminishing the toxicity of sgCas9-Adv@TCM subsequent to intravenous administration, thereby faithfully reflecting its therapeutic efficacy.

Developing effective quality control strategies for the mass production of sgCas9-Adv@TCM poses a significant challenge, but leveraging the advancements in engineered autologous cell production, it may be possible to utilize patient-derived cells as a viable source of TIGIT-overexpressing cell membranes to meet clinical standards in terms of quality and quantity. Moreover, although our initial in vivo biocompatibility assay demonstrated minimal toxicity, thorough evaluation is required to assess the risk-benefit profile of sgCas9-Adv@TCM on off-target tissues like the liver or spleen with dominant accumulation. Overall, this study proposes a promising and long-lasting therapeutic approach for cancer immunotherapy using a stealth adenoviral vector to efficiently edit the PD-L1 genome and block TIGIT/CD155, potentially addressing challenges such as low response rates, drug resistance, and immune-related adverse events associated with current combinational immune checkpoint inhibitors. Moreover, this strategy could serve as a generalizable approach for the development of systemically administered adenoviral vectors for refractory tumors that require repeated dosages.

## CRediT authorship contribution statement

**Ming Wu:** Conceptualization, Formal analysis, Funding acquisition, Investigation, Writing – original draft. **Cao Zhang:** Formal analysis, Investigation, Writing – original draft. **Aoxue Zhong:** Formal analysis, Investigation. **Hao Li:** Formal analysis, Investigation. **Yang Zhou:** Resources. **Xiaoyuan Zheng:** Resources. **Youshi Zheng:** Methodology. **Yuting Zhang:** Methodology. **Yanbing Cao:** Methodology. **Zhenli Li:** Software. **Cuilin Zhang:** Methodology. **Xiaomo Wu:** Writing – review & editing. **Dong Cen:** Conceptualization, Investigation, Project administration, Validation, Visualization. **Xiaolong Liu:** Conceptualization, Funding acquisition, Supervision, Writing – review & editing.

## Declaration of competing interest

The authors declare that they have no conflicts of interest in this work.

## Appendix Supplementary materials

Image, application 1
