# Supplementary material for: Systemic delivery of stealth adenoviral vector to reinvigorate antitumor immunity by PD-L1 genome editing and TIGIT/CD155 blocking
Source: Fundam Res. 2025 May 14;6(5):3417–30. doi: 10.1016/j.fmre.2025.04.015 (PMC13644482; doi:10.1016/j.fmre.2025.04.015)
Supplement: Supplementary file 1 [file mmc1.docx]

Supplementary Materials for

**Stealth adenoviral vector reinvigorates antitumor immunity by targeting PD-L1 and CD155 in preclinical models of hepatocellular carcinoma**

**The PDF file includes:**

Materials and Methods

Figs. S1 to S22

Table S1

**Materials and Methods**

Plasmid pX458 was purchased from Addgene (USA). Plasmid pDC315 and plasmid pBoB were purchased from Chinese Academy of Sciences. Plasmid pBHGlox (delta) E1, 3Cre was purchased from GeneBiogist Technology (Shanghai, China). The guide RNA and primers were synthesized by BIOSUNE Biological Technology (Shanghai, China). T4 DNA ligase (Cat. No. FD0054) was acquired from Thermo Fisher. T7E1 (Cat. No. M0302S) was obtained from New England Biolabs, Inc. Fetal bovine serum (FBS) was purchased from ExCell Bio (Shanghai, China).

The mouse anti-PD-L1 antibody (Cat. No. ab213480), human anti-PD-L1 antibody (Cat. No. ab213524), mouse anti-TIGIT antibody (Cat. No. ab233404), human anti-TIGIT antibody (Cat. No. ab243903), anti-β-actin antibody (Cat. No. ab179467) for western blot analysis were obtained from Abcam (UK). The mouse anti-CD155 (Cat. No. 58025T) for western blot analysis were obtained from Cell Signaling Technology (USA). The anti-TIGIT-APC antibody (Cat. No. 17-9500-42) for flow cytometry analysis were accessed from Thermo Fisher. Phenylmethylsulfonyl fluoride (PMSF) and protease inhibitor cocktail were purchased from MedChemExpress (USA). The mouse anti-PD-L1 antibody (Cat. No. 64988T) for IHC was purchased from Cell Signaling Technology. The mouse antibodies for flow cytometry analysis (anti-CD45-eFluor™ 450, anti-CD3-APC, anti-CD4-FITC, anti-CD8-PE, anti-CD25-PerCP-Cy5.5, anti-Foxp3-PE-Cy7, anti-IFN-γ-PE-Cy7, anti-CD44-PE-Cy7, anti-CD62L-PerCP-Cy5.5, anti-PD-1-PE-Cy7, Tim-3-PerCP-Cy5.5, CD69-FITC, anti-CD11c-APC, anti-CD80-PE, and CD86-PE-Cy7) were accessed from Thermo Fisher (USA). The human anti-CD8-PerCP-Cy5.5(Cat. No. 45-0088-42) and anti-IFN-γ-PE-Cy7 (Cat. No.25-7319-82) were accessed from Thermo Fisher (USA). The human anti-Granzyme B (Cat. No. 372208) were accessed from BioLegend (USA). BD Cytofix/Cytoperm^TM^ Plus Fixation/Permeabilization Kit with BD GolgiStop™ (Cat. No. 554715) and Transcription Factor Buffer Set (Cat. No. 562574) were obtained from BD Biosciences (USA). Zombie Aqua™ Fixable Viability Kit (Cat. No. 42310) was purchased from BioLegend (USA). IFN-γ, TNF-α, IL-6, and IL-10 ELISA Kits were purchased from [FANKEWEI](https://www.baidu.com/link?url=J0cl1g-DEHhNmdUDJYgJEEjd33E3NDopVOw4Zf64sOK7MP0QmyCXZGiDMCVgN1cR&wd=&eqid=83c68e2000092d160000000662c59907) (Shanghai, China). IVISBrite^TM^ D-Luciferin was obtained from PerKinElmer (USA). Mouse adenovirus antibody (IgG) ELISA Kit and Mouse TIGIT (T cell immunoreceptor with Ig and ITIM domains) ELISA Kit were purchased from CUSABIO (Wuhan, China). The mouse anti-AFP antibody (Cat. No. 14550-1-AP) and anti-GPC3 antibody (Cat. No. SC65-443) for IHC were obtained from Proteintech and Santa Cruz, respectively.

**Cell culture**

Chinese hamster ovary (CHO) cells, mouse liver cancer (Hepa 1-6) cells, human embryonic kidney (293A and 293FT) cells, human liver cancer (SK-He and SMMC-7721) cells were cultured with DMEM medium (10% FBS, 1% penicillin and streptomycin) in standard incubators (37 ℃, humidified atmosphere of 5% CO_2_). Except for the CHO cell line derived from GMP (Jena, Germany), all other cell lines were derived from ATCC (Manassas, VA).

**Construction of sgCas9 adenovirus vectors (****sgCas9-Adv)**

Briefly, the designed U6-sgRNA-CMV-Cas9-EGFP cassette (total 6301 bp) was inserted into the adenovirus shuttle plasmid (pDC315) to generate the sgCas9-Adv shuttle plasmid (pDC315-U6-sgPD-L1-CMV-Cas9-EGFP). Lipofectamine 3000 transfection reagent (Cat. No. 2098843, Thermo Fishe) was used to co-transfect the sgCas9-Adv shuttle plasmid and the adenovirus backbone plasmid of pBHGlox (delta) E1, 3Cre into 293A cells. After transfection for 3-5 days, the cells were ruptured by repeated freezing and thawing in liquid nitrogen before centrifugation at 2000 ×g for 15 minutes at 4°C to collect the supernatant containing the primary virus. The fresh 293A cells were then infected with the supernatant, and the amplified virus was purified by CsCl gradient density centrifugation and stored at -80°C.

The sgCas9-Adv genome was extracted using the Easy Pure Viral DNA/RNA Kit (TransGen Biotech, lot: Q10619) to determine the virus titer by qRT-PCR. Afterward, negative staining with uranyl acetate was performed on copper grids to observe the morphology of sgCas9-Adv using a transmission electron microscope (FEI Tecani G20 TWIN).

**Establishment of genetically engineered cell lines**

Initially, plasmids containing murine TIGIT fused with an EGFP tag at the C-terminus (pBoB-CMV-TIGIT-EGFP-puro) were constructed. Lentiviruses were generated from 293FT cells transfected with the above plasmids using the Lipofectamine 3000 Transfection Kit. Two days after infection of CHO cells with lentivirus and 5 g/mL Polybrene (Santa Cruz biotechnology, SC-134220), CHO-TIGIT cells stably expressing murine TIGIT fused to the EGFP tag were sorted by flow cytometry (BD FACS AriaTM Fusion Cell Sorter Special Order System).

Next, the cells were fixed with 4% paraformaldehyde and stained with 4′,6-diamidino-2-phenylindole (DAPI) and a membrane dye (DiI) for 10 minutes. After washing with PBS three times, the cells were observed using a confocal laser scanning microscopy (CLSM; ZEISS). Flow cytometry was also performed to verify the expression of TIGIT on cells. Additionally, the stable expression of TIGIT-EGFP in the engineered CHO cells was further characterized using a Western blot assay.

In addition, we generated human TIGIT proteins on 293A cells using lentiviral transduction from the plasmid of PCDH-TIGIT-Puro. Confirmation of their expression was achieved by staining with human anti-TIGIT antibody and analysis using both flow cytometer and Western blot assay.

**Cell membrane extraction**

The membrane from TIGIT-expressing cells was retrieved by first digesting the cells with trypsin and washing them twice with 1 × phosphate buffer solution (PBS). The cell pellets were then resuspended in lysis buffer and kept on ice for 30 minutes with gentle lysis (lysis buffer consisted of 20 mM Tris-HCl, 10 mM KCl, 2 mM MgCl_2_, and protease inhibitor). The cells were then subjected to three cycles of freeze-thawing using liquid nitrogen. The cell lysate was centrifuged at 7000g for 10 minutes, and the supernatant was collected and further centrifuged at 40,000g for 1 hour at 4 ℃. The precipitates of TCM were obtained and re-dispersed in PBS, which then stored at -80℃ for further usage. Membrane protein concentration was quantified by Easy Ⅱ Protein Quantitative Kit (BCA assay). For preparing TCM-NVs, TCM in PBS solution were treated with 200 W ultrasound for 2 min, following 21 cycles of extrusion through a polymeric membrane (0.8 μm) using an extruder (Bleb-derived Giant Vesicles).

**Cell membrane encapsulation of sgCas9 Adenovirus (****sgCas9-Adv@TCM)**

Similar to the preparation of TCM-NVs, sgCas9-Adv@TCM was prepared using sgCas9-Adv together with cell membrane by extrusion through a polymeric membrane 21 times in an extruder. The TCM were completely suspended in an ice water bath at 200 W. Ultrasonic treatment was performed for 2 min when the TCM became slightly transparent. The TCM were then mixed with sgCas9-Adv at a ratio of 300 μg cell membranes: 2×109 viral particles based on protein weight and virus titer, respectively. The system was then extruded 21 times through a polymeric membrane (0.8 μm) using an extruder.

**Western blot**

Cell lysates were prepared in RIPA lysis buffer supplemented with PMSF and protease inhibitor cocktail on ice for 30 minutes, and the protein concentration of the resulting lysates was determined using a BCA protein assay. Subsequently, each sample was mixed with loading buffer and heated to 100°C for 10 minutes before being loaded onto a 10% SDS-PAGE gel. The gels were run at 110 V for 2 hours and then transferred onto nitrocellulose (NC) membranes. After blocking with 5% BSA for 1 hour, the NC membranes were incubated overnight at 4°C with specific primary antibodies, including TIGIT, PD-L1, and β-actin antibodies. Next, the membranes were washed with 1× TBST and then incubated with HRP-conjugated corresponding secondary antibodies (1:5000) for 1 hour at room temperature. Finally, the membranes were visualized using SuperSignal^TM^ West Pico PLUS Chemiluminescent Substrate and recorded using the ChemiDoc^TM^ MP imaging system.

**Immunoprecipitation (IP) assay**

The orientation of TIGIT on the TCM-NVs was confirmed as follows: TCM-NVs were dispersed in 1 mL ice-cold PBS and incubated for 2 h at 4 °C with gently rotating beads made up of 50% protein A/G agarose. This mixture was then centrifuged at 10,000 rpm for 15 minutes at 4 °C to precipitate TCM-NVs that were nonspecifically bonded to protein A/G agarose beads. Next, the supernatant of the TCM-NVs was incubated overnight at 4°C with TIGIT antibody and protein A/G agarose beads. The beads were then washed 3 times with ice-cold PBS. Finally, western blot analysis was conducted with the appropriate antibodies after the samples were subjected to 10% SDS-PAGE. IgG was used as a substitute for the TIGIT primary antibody in the control group.

**Zeta (ζ)-potential and dynamic light scattering analysis**

The TCM-NVs, naked sgCas9-Adv, and sgCas9-Adv@TCM were vortexed and diluted with sterile milli-Q water to a final volume of 1 mL. The size was determined using a Zetasizer Nano ZS (Malvern) in a polystyrene disposable cuvette at 25 °C. Subsequently, the sample was recovered from the cuvette and transferred to a disposable capillary cell for further zeta potential measurements.

**Coomassie blue assay**

The samples of sgCas9-Adv, TCM-NVs, and sgCas9-Adv@TCM were subjected to SDS-PAGE after being heated at 100 °C for 10 minutes in the loading buffer. A 10% SDS-PAGE gel was used for separation, followed by staining with Coomassie blue for 30 minutes. Subsequently, the gel was destained using a solution consisting of 40% ethanol, 10% acetic acid, and 50% deionized water for 3 hours, and an image of the gel was captured using the imaging system (ChemiDoc^TM^MP, Bio-Rad).

**Cell binding assay**

1.5×10^5^ Hepa 1-6 cells were seeded in confocal dishes and cultured to allow cell adhere. Transfection of pDC315-MCMV-CD155-mCherry-puro plasmid into the cells was achieved through the use of Lipofectamine 3000 Transfection Kit. After 24 h, the medium was changed to a fresh medium containing TCM-NVs (50 μg/ml based on total protein content) labeled with DiO and incubated with Hepa 1-6 cells at 4°C for 1 h. After washing the cells with PBS, the cells were fixed with 4% paraformaldehyde for 15 minutes and stained with DAPI for 10 minutes. Lastly, the cells were washed 3 times with PBS before being observed with a CLSM (Zeiss LSM780).

**Cellular uptake assay**

1.5×10^5^ Hepa 1-6 cells were seeded in confocal dishes and cultured to allow cell adhere, and then the TCM-NVs or CM-NVs (50 μg/mL based on protein content) labeled with DiI were incubated with Hepa1-6 cells for 6 h at 37 °C. After washing the cells with PBS, the cells were fixed with 4% paraformaldehyde for 15 minutes and stained with DAPI for 10 minutes. Lastly, the cells were washed 3 times with PBS before being observed with a CLSM (Zeiss LSM780).

**Transduction of sgCas9-Adv@TCM**

An evaluation of the cellular infection efficiency of sgCas9-Adv@TCM was conducted using EGFP fluorescence. Typically, the Hepa1-6 cells were inoculated in 6-well plates one day before infection with the sgCas9-Adv and sgCas9-Adv@TCM (10^7^ viral particles per well for sgCas9-Adv, 50 μg/mL per well for TCM based on protein content), and then the cellular EGFP fluorescence was assessed using the fluorescence microscopy (Zeiss Axio Vert.A1, Germany) after 48 h of infection.

**Knockout of cellular PD-L1 by** **sgCas9-Adv@TCM**

The knockout effect of sgCas9-Adv@TCM on PD-L1 was evaluated by CLSM, western blot, T7 endonuclease I (T7E1) assays and Sanger sequencing, respectively. In brief, Hepa1-6 cells infected with sgCas9-Adv@TCM for 48 h were fixed for 15 min with 4% paraformaldehyde, then incubated for 4 h with anti-PD-L1 antibody (abcam, ab213480), followed by goat anti-rabbit IgG for 2 h and stained with DAPI for 10 min (the cells were washed 3 times with PBS between staining steps). Lastly, the cells were observed with a CLSM.

The PD-L1 knockout effect was also confirmed by western blot. Hepa1-6 cells that were infected with sgCas9-Adv@TCM for 48 hours were collected, then treated by RIPA lysis buffer supplemented with PMSF and protease inhibitor cocktail on ice for 30 minutes as indicated above. Specially, anti-PD-L1 antibody (1:1000) and anti-β-actin antibody (1:5000) were used as primary antibodies, along with HRP-conjugated secondary antibodies (1:5000) for western blot analysis.

For T7 endonuclease I (T7E1) assays and Sanger sequencing, the experimental procedure involved infecting Hepa1-6 cells for 48 hours, followed by extraction of cellular DNA using the EasyPure Viral DNA/RNA Kit (TransGen Biotech, lot: Q10619). To analyze indels, PCR primers were designed to amplify a region approximately 300 bp upstream and 700 bp downstream of the PD-L1 gene. The resulting PCR products were digested with T7 Endonuclease I (T7E1), separated by 1% agarose gel electrophoresis, and visualized using an imaging system. Indel efficiency was calculated using the formula [(b+c)/(a+b+c) × 100%], where a is the uncut band around 1000 bp, and b and c are cut bands around 300 bp and 700 bp, respectively. Finally, Sanger sequencing was performed on the PCR products to provide additional information.

**Antibody neutralization assay**

All animal procedures were reviewed and approved by the Animal Ethics Committee of the Mengchao Hepatobiliary Hospital of Fujian Medical University in accordance with the "National Animal Management Regulations of China". To determine neutral antibody levels in vivo, C57BL/6 mice were intravenously injected with sgCas9-Adv@TCM (equivalent to 2×109 viral particles per mouse for sgCas9-Adv and 300 μg per mouse for TCM-NVs). Mice received a total of 4 intravenous injections every 3 days, and blood was collected from the tail vein 18 days after the last injection. The level of neutralizing antibodies in the blood was measured using a mouse adenovirus antibody (IgG) ELISA Kit (CSB-E13901m, CUSABIO).

To assess the resistance of sgCas9-Adv@TCM to neutralizing antibodies, serum was allowed to stand at room temperature for 15 minutes before centrifugation at 4°C. The resulting serum was then diluted with culture medium at ratios of 1:0, 1:100, and 1:2000 and incubated with either sgCas9-Adv or sgCas9-Adv@TCM at 37°C for 1 hour. The mixture was then transferred to Hepa1-6 cells, and the resulting intracellular EGFP fluorescence was observed by fluorescence microscopy and flow cytometry, respectively.

To further confirm the resistance of sgCas9-Adv@TCM to neutralizing antibodies, mice were intravenously injected with sgCas9-Adv or sgCas9-Adv@TCM at 0, 24, 48, and 72 hours, and the blood was collected from the tail vein at 24, 48, 72, and 96 hours. Adenovirus genome copies were determined by qRT-PCR after extracting genomes from blood with the QIAamp DNA Mini Kit (QIAGEN, Cat. No. 51306).

**In vivo distribution of sgCas9-Adv@TCM**

To investigate the distribution of sgCas9-Adv@TCM in tumor-bearing mice, Hepa1-6 tumors were established by subcutaneously injecting 3×10^6^ murine Hepa1-6 cells in 100 μL of a sterile PBS/Matrigel mixture (1:1, v/v) into the armpit of C57BL/6 mice. When the tumor grew to 200 mm^3^, the mice were injected intravenously with DiR-labeled sgCas9-Adv@CM or sgCas9-Adv@TCM (equivalent to 2×10^9^ viral particles per mouse for sgCas9-Adv and 300 μg per mouse for TCM). The mice were then imaged by bioluminescence imaging system (PerkinElmer IVIS Spectrum, MA, USA) 48 hours after administration.

**In vivo Analyses of biosafety**

After 48 h of intravenously administering sgCas9-Adv@TCM (equivalent to 2×10^9^ viral particles per mouse for sgCas9-Adv and 300 μg per mouse for TCM) to healthy C57BL/6 mice, the mice were euthanized and their blood, liver, and spleen were collected to evaluate the safety of sgCas9-Adv@TCM. The serum was prepared as previously described before measuring biochemical parameters, and paraffin sections of a portion of the liver and spleen were stained with H&E.

To assess the impact of sgCas9-Adv@TCM on immune response in the liver and spleen, 20-50 mg of liver and spleen samples were collected and lysed in RIPA buffer (Beyotime Biotechnology, China) containing protease inhibitor cocktail (MedChemExpress, USA). The lysates were then homogenized with magnetic beads at 60 Hz for 180 s, followed by centrifugation at 16,000×g for 10 min at 4°C. The levels of IL-6 and TNF-α in the supernatants were analyzed by ELISA Kits (FANKEWEI, China). Additionally, the remaining liver and spleen samples were digested (using 1 mg/mL of collagenase Ⅳ, 0.2 mg/mL of hyaluronidase, and 0.02 mg/mL of Dnase I for 2 h at 37℃) to form a single-cell suspension. After centrifugation at 800 g for 5 minutes, the single-cell suspension was further purified using Ficoll-Paque density gradient centrifugation, and then the cells were stained with anti-CD3-APC, anti-CD4-FITC and anti-CD8-PE and analyzed using flow cytometry.

To analyze virus-induced Kupffer cell death in vivo, mice were injected intravenously with different viruses (2 × 10⁹ viral particles per mouse). 50 minutes after the virus injection, 50 μg of propidium iodide (PI) was administered in 200 μL of PBS. 10 minutes post-PI injection, the mice were euthanized. Livers were harvested, embedded in OCT compound, and frozen for preservation. Liver sections were subsequently prepared, fixed, and stained with anti-CD68-AF647 (Cat. No. 137003, BioLegend) to identify Kupffer cells.

**Anti-tumor on subcutaneous HCC model**

The Hepa1-6 subcutaneous tumor model was constructed as described above. Once the tumors had grown to an average size of approximately 100 mm^3^, the mice were divided into five groups: PBS, sgCas9-Adv, sgCas9-Adv@CM, sgCas9-Adv + TCM-NVs, and sgCas9-Adv@TCM. Each mouse received 2×10^9^ viral particles of sgCas9-Adv, while TCM were administered at a dose of 300 μg per mouse. The injections were given intravenously in a volume of 200 μL, and a total of four doses were given at three-day intervals. Tumor volume and mouse weight were monitored throughout the treatment period. If a mouse's health deteriorated or if its tumor volume exceeded 1500 cm^3^, it was euthanized. Tumor volumes were calculated using the formula V=a×b^2^/2, where a and b represent the maximum and minimum diameters measured with vernier calipers, respectively.

To further investigate the role of T cell populations in inhibiting tumor growth, tumor-bearing mice were intraperitoneally injected with 100 μg of anti-CD4 antibody (Cat. No. BE0003-1, Clone: GK1.5, BioXCell) or anti-CD8 antibody (Cat. No. BE0117, Clone: YTS 169.4, BioXCell) one day prior to each virus treatment. A control group of mice received 100 μg of IgG isotype antibody (Cat. No. BE0090, Clone: LTF-2, BioXCell) under the same conditions.

**Anti-tumor on orthotopic HCC model**

To evaluate the effectiveness in inhibiting orthotopic Hepa1-6 tumor growth, an equal volume of Matrigel suspension was mixed with 4×10^5^ Luc-Hepa1-6 cells (25 μL) and injected gradually into the liver lobe via a surgical operation. Afterwards, 150 μL IVISBrite^TM^ D-luciferin (20 mg/mL) was injected intraperitoneally on day 7 to confirm the establishment of the orthotopic Hepa1-6 tumor model, through an in vivo fluoresence imaging system (UNITED WELL) equipped with an Andor CCD camera. Then, the mice were randomly divided into five groups: PBS, Anti-PD-L1 (100 μg per mouse by i.p.), Anti-TIGIT (100 μg per mouse by i.p.), Anti-(PD-L1+TIGIT) (100 μg of each antibody per mouse by i.p.), sgCas9-Adv@TCM. A total of 4 doses were administered to mice every 3 days. The growth of orthotopic Hepa1-6 tumors was reflected by the average radiance calculated from the bioluminescence signals (photons, s^−1^ cm^−2^ sr^−1^).

**Anti-tumor effect evaluated by histological evaluation**

Tumors were collected from mice on day 4 after the last administration, then made into frozen sections or paraffin sections for H&E, Ki67, TUNNEL, immunofluorescence or immunohistochemical staining.

**Flow cytometric analysis**

Spleen was collected to flush out splenic cells by a sterile 5 mL syringe. Tumor draining lymph node (TDLN) were minced and meshed into a single-cell suspension using the back side of syringe plunger. Liver and tumor tissues were minced into fine pieces and dissociated into single-cell suspensions with collagenase type Ⅳ (1 mg/mL), hyaluronidase (0.2 mg/mL), DNase Ⅰ (0.02 mg/mL) at 37°C for 2 hours. The cells were then isolated through a 40 μm cell strainer and Ficoll-Paque density gradient centrifugation at 800 g for 20 min. Next, the cell suspensions were incubated with the Zombie Aqua™ Fixable Viability Kit to assess cell viability. Following this, the cells were treated with an anti-CD16/CD32 Fc receptor (FcR) block to prevent nonspecific antibody binding. The samples were then stained with various panels of fluorophore-conjugated antibodies targeting surface markers. For subsequent intracellular staining, such as IFN-γ, the cells were pre-stimulated with the cell stimulation cocktail (Cat. No. 00-4975-93, Thermo Fisher) at 37°C for 6 hours and fixed and permeabilized using the BD Cytofix/Cytoperm^TM^ Plus Fixation/Permeabilization Kit with BD GolgiStop^™^ before stained with IFN-γ and other surface antibodies. For transcription factor staining, such as Foxp3, cells were fixed, permeabilized and washed using Transcription Factor Buffer Set according to the manufacturer's protocol. Finally, the stained cells were analyzed by BD FACSVerse^TM^ (BD Biosciences) or NovoCyte Advanteon Dx (Agilent). Markers used for different cell lineages were:

CTL (Cytotoxic T Lymphocytes): CD45^+^CD3^+^CD8^+^

Effector CTL: CD45^+^CD3^+^CD8^+^ IFN-γ^+^

Exhaust CTL: CD45^+^CD3^+^CD8^+^PD-1^+^Tim-3^+^

Activated CTL: CD45^+^CD3^+^CD8^+^CD69^+^

Th (Helper T Lymphocytes): CD45^+^CD3^+^CD4^+^

Treg: CD45^+^CD3^+^CD4^+^CD25^+^Foxp3^+^

**Anti-tumor efficacy on subcutaneous CD155-knock-out (CD155-KO) HCC model**

CD155 knockout (KO) Hepa 1-6 cells were generated using CRISPR/Cas9 genome editing via tube electroporation. The electroporation equipment, including the machine, electroporation tubes, and buffer, was provided by Celetrix. A total of 1 × 10⁶ Hepa 1-6 cells were resuspended in 25 μL of electroporation buffer. For Cas9 ribonucleoprotein (RNP) transfection, 1 μg of Cas9-NLS protein was pre-incubated with 3 μg of sgRNA (previously reported to target CD155 gene loci, CAAGACGCCTGTCGAATTGT/ CTGGTGCCCTACAATTCGAC) for 15 minutes to form the RNP complex. The RNP complex was then mixed with the cell suspension and transferred into an electroporation tube. Electroporation was performed at 540 V with a pulse duration of 30 ms. Following electroporation, the cells were immediately transferred into warm culture medium and incubated for 48 hours. CD155 expression was subsequently analyzed by western blotting.

Subsequently, CD155 KO Hepa 1-6 cells were used to establish a subcutaneous HCC model to evaluate the therapeutic efficacy of sgCas9-Adv@TCM.

**RNA sequencing and bioinformatic analysis**

The RNA library was prepared using the VAHTS Stranded mRNA-seq Library Prep Kit for IlluminaV2 kits based on RNA samples extracted from the tumor tissues after receiving indicated treatments. RNA-seq was then performed at Fulgent. Co. Ltd. using an Illumina Navoseq 6000 platform.

With the help of STAR, all qualified raw reads were aligned to the mouse genome (mm10, https://www.gencodegenes.org), and FPKM (transcripts per million) was further used to quantify gene expression levels. Cufflinks v2.2.1 was used to analyze differential expression between different groups, and clusterProfiler package was used to analyze KEGG pathway enrichment using differentially expressed genes (|log_2_ fold change| > 1, p value < 0.05).

**Preparation of autogenous CD8^+^ T cells**

PBMCs were obtained from patients using Ficoll-Hypaque gradient separation. Primary human CD8^+^ T cells were subsequently isolated with a negative purification method employing magnetic bead with the enrichment set DM (BD-IMag). T lymphocytes were then stimulated using anti-CD3/CD28 Dynabeads (Miltenyi Biotec, Germany) and cultured in KBM 581 medium (Corning, NY, USA) supplemented with recombinant IL-2 (10 ng/mL) (R&D Systems). Cell feeding was conducted twice weekly to sustain the cell concentration at 1 × 10^6^ cells/mL, utilizing KBM581 medium supplemented with IL-2 (10 ng/mL).

**Anti-tumor study on PDO models**

Briefly, tumor tissues from patient I were dissected into small pieces and incubated in a digestion solution with gentle agitation. Termination of the digestion process was achieved by adding 10% fetal bovine serum (FBS) when most of the tissues are in a single-cell state after 10-20 minutes. Subsequently, the suspension underwent filtration using a 100 μm strainer, followed by centrifugation for 3 minutes at 300g. Then, the supernatant was discarded, and the pellet underwent two washes. The obtained organoids were then embedded in Matrigel (Corning). Upon solidification of the Matrigel, the cells were overlaid with PDO culture medium (biogenous, K2105-HCC). Afterwards, the organoids were passaged after dissociation with Organoid Dissociation Solution (biogenous, E238001). For storage purposes, the organoids were resuspended in Recovery-cell-culture freezing medium (GIBCO) and frozen using standard procedures.

To assess the therapeutic efficacy of sgCas9-Adv@TCM on PDOs, the organoids were isolated from Matrigel and seeded into a 96-well plate. Subsequently, they were infected with sgCas9-Adv@TCM for 48 hours. Afterwards, the PDOs were co-cultured with autologous CD8^+^ T cells at an effector-to-target ratio (E:T) of 10:1 for an additional 48 hours. To determine the E:T ratio, a portion of the organoids was dissociated into single cells for counting. Finally, the PDOs were stained with a red fluorescent caspase3/7 probe (C10430, Invitrogen) that binds to DNA upon cleavage by caspase3/7 for 45 minutes and subsequently imaged using CLSM.

**Anti-tumor study on PDX models**

We used the 6-week-old NCG mice (NOD/ShiLtJGpt-Prkdc^em26Cd52^Il2rg^em26Cd22^/Gpt) , supplied by Guangdong Gempharmatech Co., Ltd to build PDX models. Berifly, the tumor samples from patient II were cutted into small pieces (2×2×2 mm³), and a portion of them were engrafted in NCG mice for three passages; the PDX samples were then subcutaneously transplanted into the axilla of the mice to establish the PDX models. To evaluate the therapeutic efficacy, each mouse was injected either i.t. with sgCas9-Adv@TCM (100 μg in 100 μL PBS) or i.p. with combinational human anti-PD-L1 (Cat. No. A2004, Selleck) and anti-TIGIT (Cat. No. A2028, Selleck) (100 μg of each antibody in 100 μL PBS) at day 0 and day 4, while 1 × 10^6^ autologous CD8^+^ T cells mixing with IL-2 (1000 IU) were intravenously infused into the mice on days 2 and 6. The mice only i.t. injected with PBS or without CD8^+^ T cell infusion was used as the controls.

To study the immune state of tumor infiltrating CD8^+^ T cells, single-cell suspension was directly stained with human anti-CD8a-PerCP-Cy5.5 (Cat. No. 45-0088-42, Thermo Fisher), human/mouse anti-IFN-γ-PE-Cy7 (Cat. No. 25-7319-82, Thermo Fisher), and human/mouse anti-Granzyme B-PE (Cat. No. 372208, BioLegend) for flow cytometric analysis.

**Statistical Analysis**

One-way analysis of variance (one-way ANOVA), two-way analysis of variance (two-way ANOVA) or unpaired two-tailed Student's t test were performed using the statistical analysis software GraphPad Prism 8.0. The survival rate was compared using the log-rank test. Differences between the groups were considered statistically significant at *P<0.05, **P<0.01, ***P<0.001, ****P<0.0001.

**Supplementary Figures**

Figure S1. The information of pBoB-CMV-TIGIT-EGFP-puro.

Figure S2. Representative flow cytometry gating strategies for experiments in Figure 1d.

Figure S3. Full gel scan images of Figure 1e.

Figure S4. Full gel scan images of Figure 1f.

Figure S5. The information of pDC315-U6-sg-CMV-Cas9.

Figure S6. Standard curve for the determination of titer of sgCas9-Adv.

Figure S7. Representative flow cytometry gating strategies for experiments in Figure 2g.

Figure S8. Full gel scan images of Figure 2h.

Figure S9. Representative flow cytometry gating strategies for experiments in Figure 3c.

Figure S10. Original fluorescence imaging of the entire mouse body and major organs, corresponding to the data presented in Figure 3f-h.

Figure S11. Histological H&E staining of major organs after indicated treatments. Scale bar: 50 μm.

Figure S12. Serum biochemical parameters of AST and ALT, corresponding to the data presented in Figure 3j.

Figure S13. Pro-inflammatory factors of IL-6, TNF-α, IL-10, and IFN-γ in liver and spleen, corresponding to the data presented in Figure 3k.

Figure S14. Representative flow cytometry gating strategies for experiments in Figure 3l. Statistical significance was calculated by one-way ANOVA with Tukey’s multiple comparison test. ns means no significant difference.

Figure S15. Representative immunofluorescent staining of liver sections showing in vivo necrosis as PI permeability (red) of CD68-positive Kupffer cells (green) after administration of the indicated viruses to mice. DAPI staining of the nuclei is in blue. White arrows point to the PI-positive necrotic Kupffer cells.

Figure S16. Photograph of Hepa1-6 tumor-bearing mice after receiving different treatments as indicated. The tumor area was labeled with a red circle.

Figure S17. Statistical analysis of three fields from Ki67 and TUNEL images, as represented in Figure 4h. Statistical significance was calculated by one-way ANOVA. ****P<0.0001.

Figure S18. Therapeutic efficacy of sgCas9-Adv@TCM on CD155-KO Hepa1-6 subcutaneous tumors. (a) Western blot of CD155 protein expression of CD155-KO Hepa1-6 cells. (b) Average tumor volume and photographs after receiving sgCas9-Adv@TCM treatment (n = 5). Data in (b) are presented as mean ± SD. Statistical significance was calculated by two-tailed Student’s t-test. ***P<0.001.

Figure S19. Full gel scan images of Figure 5c.

Figure S20. Representative flow cytometry gating strategies for experiments in Figure 5d. To detect the percentage of CD3^+^CD4^+^ T cells, CD3^+^CD8^+^ T cells, Treg cells, and IFNγ^+^CD8^+^ T cells in the tumor.

Figure S21. The percentage of tumor-infiltration of PD-1^+^ TIM-3^+^ T in CD8^+^ T cells after receiving different treatments, which were analyzed by flow cytometry on the 4th day (n = 3). Data are presented as mean ± SD. Statistical significance was calculated by one-way ANOVA with Tukey’s multiple comparison test. ****P<0.0001.

Figure S22. Cytokine levels of IFN-γ and TNF-α in tumor lysates by ELISA analysis after indicated treatments (n=3). Statistical significance was calculated by one-way ANOVA. **P<0.01, ****P<0.0001.

Figure S23. Therapeutic efficacy of sgCas9-Adv@TCM upon the depletion of CD8^+^ T or CD4^+^ T in Hepa1-6 subcutaneous tumors (n = 4 animals per group). Statistical significance was calculated by one-way ANOVA. *P<0.05, ****P<0.0001.

Figure S24. Bioluminescence imaging of mice in each group every one week as indicated.

Figure S25. Average tumor growth (measured by total radiance) kinetics in different groups (n = 6 per group). Statistical significance was calculated by two-way ANOVA. ****P<0.0001.

Figure S26. Immunohistochemistry staining of tumor infiltrated CD8^+^ T cells on the day 105 after receiving different treatments.

Figure S27. The percentage of tumor-infiltration of CD69^+^CD8^+^ T cells after receiving different treatments (n = 3). Data are presented as mean ± SD. Statistical significance was calculated by one-way ANOVA with Tukey’s multiple comparison test. ****P<0.0001.

Figure S28. Representative flow cytometry gating strategies for experiments in Figure 6h. To detect the percentage of central memory T cells and effective memory T cells in spleen.

Figure S29. The levels of IFN-γ and IL-10 in peripheral blood after receiving different treatments as indicated in Figure 6i. Data are presented as mean ± SD. Statistical significance was calculated by one-way ANOVA with Tukey’s multiple comparison test. **P<0.01, ***P<0.001.

Figure S30. Preparation and characterization of humanized sgCas9-Adv@TCM. (a, b) Infectivity of Cas9 Adv, sg-1 Cas9-Adv and sg-2 Cas9Adv on SK-Hep and SMMC-7721 cells analyzed by fluorescence microscopy (a) and flow cytometry (b) (n = 3). (c) Western Blot of PD-L1 protein expression of SK-Hep and SMMC-7721 cells after infection with Cas9 Adv, sg-1 Cas9-Adv and sg-2 Cas9-Adv. (d, e) The T7E1 assay(d) and Sanger sequencing(e) of PCR amplicon of the targeted PD-L1 locus in SMMC-7721 cells after transduction with sg-2 Cas9-AdV. (f) The design of TIGIT-encoding plasmid to construct 293A-TIGIT cells. (g) The representative ﬂow cytometric analysis of 293A-TIGIT cells by flow cytometry and positive ratio statistics (n = 3). (h) Verification of TIGIT expression in 293A-TIGIT cells by Western Blot. Data in (b) and (g) are presented as mean ± SD. Statistical significance was calculated by one-way ANOVA with Tukey’s multiple comparison test in (b) and two-tailed Student’s t-test in (g). ****P<0.0001.

Figure S31. Statistical analysis of three fields for fluorescence images depicting EGFP protein expression, as represented in Figure 7c. Statistical significance was calculated by one-way ANOVA with Tukey’s multiple comparison test. ***P<0.001.

Figure S32. Statistical analysis of three fields from immunofluorescence images depicting PD-L1 protein expression, as represented in Figure 7d. Statistical significance was calculated by one-way ANOVA with Tukey’s multiple comparison test. ***P<0.001.

Figure S33. Statistical analysis of three fields for CLSM images of PDOs stained with caspase 3/7 probe, as represented in Figure 7e. Statistical significance was calculated by one-way ANOVA with Tukey’s multiple comparison test. ***P<0.001.

Figure S34. Histological sections of primary HCC tissues and PDOs stained with H&E. AFP, GPC3, CD155, and PD-L1 were detected by immunohistochemistry. Scale bar: 50 μm.

Figure S35. Representative flow cytometry analysis of tumor-infiltration of CD8^+^ T, IFN-𝛾^+^CD8^+^ T and Granzyme B^+^CD8^+^ T cells after receiving different treatments. Related to Figure 8h.

Table S1. HCC Patient information
